# NIR light-driven nanomotor with cascade photodynamic therapy for MRSA biofilm eradication and diabetic wound healing

**DOI:** 10.7150/thno.109356

**Published:** 2025-02-24

**Authors:** Yuanyuan Deng, Jia Zheng, Jianghua Li, Bo Liu, Ke Chen, Yiling Xu, Liu Deng, Huixia Liu, You-Nian Liu

**Affiliations:** 1 Department of Geriatric Endocrine, Xiangya Hospital, Central South University, Changsha, Hunan 410083, China.; 2 College of Chemistry and Chemical Engineering, Central South University, Changsha, Hunan 410083, China.

**Keywords:** NIR light-driven nanomotor, cascade photodynamic therapy, MRSA biofilm eradication, diabetic wound healing

## Abstract

**Background:** Diabetic wounds infected with methicillin-resistant *Staphylococcus aureus* (MRSA) are challenging to heal due to biofilm formation, which impairs conventional antibiotics with limited penetration and severe side effects. Near-infrared (NIR)-driven nanomotors with autonomous motion and photothermal effects show promise for antibacterial therapy but often lack targeted specificity. Lysostaphin (Ly), an enzyme targeting bacterial cell walls, offers excellent potential against drug-resistant MRSA.

**Methods:** A novel NIR-driven CSIL nanomotor has fabricated by co-loading indocyanine green (ICG) and lysostaphin onto spinous yolk-shell structured C/SiO_2_@C nanoparticles. The autonomous motion, biofilm penetration, and antibacterial efficacy of CSIL nanomotors are evaluated *in vitro*, while their biofilm eradication and wound healing performance are assessed in an MRSA-infected diabetic mouse model using a cascade photodynamic therapy (CPDT) strategy.

**Results:** CSIL nanomotors exhibit photothermal and photodynamic properties with MRSA-targeting specificity. They can effectively eradicate MRSA biofilms both *in vitro* and *in vivo*, suppress virulence and biofilm-related genes, thus promoting diabetic wound healing by shaping a microenvironment dominated by M2 macrophages. The CPDT strategy is able to avoid excessive ROS production and thermal damage, enabling safe and effective therapy.

**Conclusion:** CSIL nanomotors, with integrated photothermal, photodynamic, and MRSA-targeting properties, represent a novel, efficient and targeted approach to antibacterial therapy in diabetic wounds, offering significant advantages over conventional antibiotics.

## Introduction

Diabetes is one of the main diseases threatening human health. About 20% of diabetes patients suffer from chronic wounds, which greatly increases the risk of death for patients 1, 2. Methicillin resistant *Staphylococcus aureus* (MRSA) has become a refractory strain of diabetes wound infection due to its colonization tendency, high invasion and strong drug resistance 3, 4. Another major obstacle to wound healing in chronic diabetes is the formation of biofilm, which is an important factor leading to bacterial drug resistance and the first line of defense for bacteria 5-8. As physical barriers, the biofilms protect the bacteria from phagocyte attacks, and prevent the penetration of antibiotics, thus resulting in insufficient sensitive to therapeutic drugs. Conventional antibiotic treatment for MRSA infections may result in high costs and adverse reactions. Recently, antibacterial proteins, such as bacteriocins 9, bacteriophage lytic enzymes 10, and bacterial autolysins 10 have received widespread attention owing to their significant efficacy in destroying bacterial cell walls. Lysostaphin exhibits high therapeutic activity against multidrug-resistant strains, including MRSA 11, 12. However, the clinical application of lysostaphin is often limited by its enzyme degradation, immunogenicity, bioavailability and adverse effects 12-14. Therefore, there is an urgent need for a treatment method with biofilm permeability and antibacterial activity to treat MRSA diabetes wound healing.

Photodynamic therapy (PDT) has made significant progress in antibacterial applications 15-17. Indocyanine green (ICG) is a NIR photosensitizer that exhibits great potential for PDT due to its high ROS generation ability. It is worth noting that ROS is a double-edged sword in antibacterial applications 18. Moderate ROS can achieve antibacterial effects and promote angiogenesis without damaging host tissues. On the other hand, ROS can cause oxidative stress and inflammatory reaction in diabetes wounds, which delays the healing of chronically infected tissues. To mitigate the risk of excessive ROS causing secondary damage to diabetes mellitus (DM) wounds, especially during the application of NIR-driven nanomotors. For example, Wang *et al.* regulated ROS production by controlling the duration of light exposure during treatment 19. Furthermore, by mitigating excessive ROS production, macrophage polarization was shifted from the M1 to the M2 phenotype, thereby accelerating the transition from the inflammatory phase to the proliferative phase 20, 21. In addition, free ICG is prone to decomposition, resulting in low PDT efficiency 22, 23. Therefore, loading and transporting ICG into tissues and precisely regulating ROS production are crucial for antibacterial and wound healing.

Nanomotors have raised great interest owing to their ability to convert chemical fuels or external stimuli, such as sound, light and electricity, into propulsive forces. The characteristic self-propulsion property endows the nanomotors with exceptional tissue penetration ability and drug delivery efficiency, thereby presenting promising prospects for broad antibacterial applications 24, 25. Among the nanomotors, Janus nanoparticles with yolk-shell structures, can combine various components into a single carrier, thus exhibiting the synergistic antibacterial therapeutic performance 26, 27. For photothermal nanomotor, when exposed to NIR, its asymmetric nanostructure can be unevenly heated and move along the temperature gradient direction 24, 28, 29. Meanwhile, the cavities between yolk and shell offer sufficient space for drug loading 30.

**Scheme 1:**
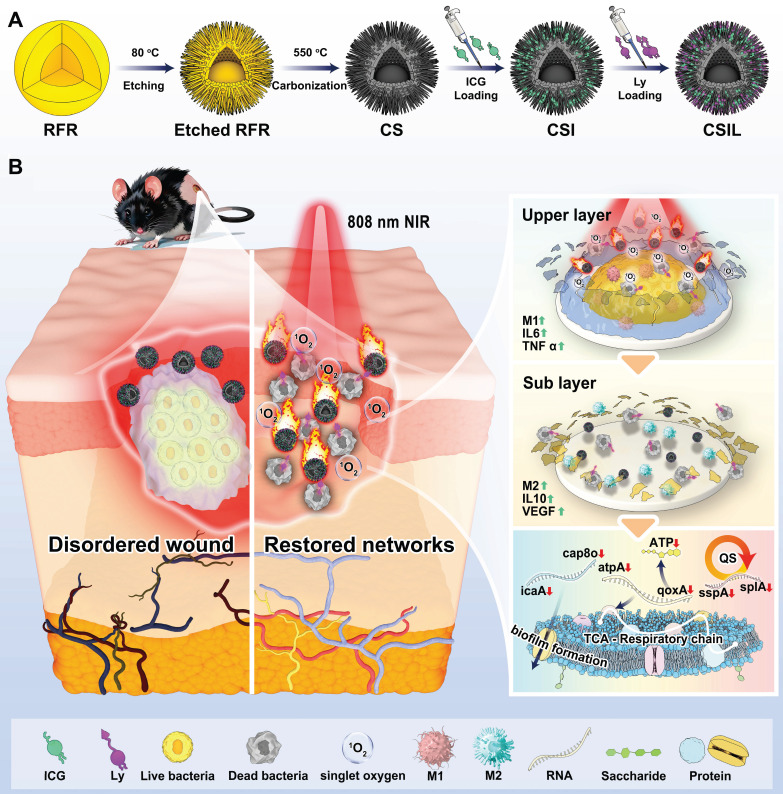
(A) Illustration of the preparation of the nanomotor with co-loading ICG and lysostaphin (CSIL). (B) Schematic diagram of NIR light-driven nanomotor for eradicating MRSA biofilm and promoting wound healing of diabetes through cascade photodynamic therapy (CPDT) strategy.

The potential applications of nanomotors in anti-biofilm encourage us to develop a strategy that allows for delivery of sufficient ICG and lysostaphin to eradicate the MRSA biofilm, which can penetrate biofilms and generate ^1^O_2_ to achieve synergistically antibacterial effects and reduce side effects. Herein, a NIR light-driven nanomotor (named CSIL) composed carbon yolk with eccentric structure and spinous shell, loaded with ICG and lysostaphin is designed for eradication of MRSA biofilms and healing wound through cascade photodynamic strategy (**Scheme 1**). In addition, CSIL nanomotor can suppress the bacterial quorum sensing system (QS) and promote the transition of macrophages from M1 (pro-inflammatory phenotype) to M2 (anti-inflammatory phenotype) polarization states during the wound healing process. Our results provide a promising new tool and strategy for the clinical treatment of diabetes wounds with MRSA infection.

## Materials and Methods

### Preparation of yolk-shell structured C/SiO_2_@C nanoparticles

Spinous yolk-shell structured C/SiO_2_@C (CS) nanoparticles were prepared according to the previous reported procedures with a slight modification 31, 32. Briefly, a mixture solution containing water (20 mL), anhydrous ethanol (140 mL), and NH_3_ aqueous solution (28 wt%, 6.2 mL) was stirred at 25 °C for 5 h, then tetraethyl orthosilicate (TEOS, 0.6 mL) was added and stirred for 10 min. After that, resorcinol (0.8 g) and formaldehyde (1.12 mL) were added separately, stirred for 2 h. The precipitate was filtrated, and washed with 50% ethanol for three times. Subsequently, the solid products were vacuum dried at 60 °C overnight, to obtained RFR/SiO_2_@RFR nanospheres. The nanospheres have a core-shell structure, where the core is composed of resorcinol formaldehyde resin (RFR) and the shell is a mixture of SiO_2_ and RFR. Resorcinol formaldehyde resin can be dissolved controllably through oxidative dissolution method 33. The as-prepared RFR/SiO_2_@RFR (200 mg) was added to Al(NO_3_)_3_ aqueous solution (2.0 mol L^-1^, 100 mL), followed by transferring to a hydrothermal reactor, reacted at 80 °C for 2 h. Then, the obtained mixture was cooled to room temperature, centrifuged, washed three times with water, and vacuum dried at 40 °C for 10 h to obtain partially etched RFR/SiO_2_@RFR nanospheres. Afterwards, the nanosphere was placed to a tube furnace and carbonized at 550 °C for 2 h in N_2_ atmosphere. Finally, the yolk-shell structured CS nanoparticles were obtained.

### Co-loading of ICG and lysostaphin on CS

To improve the loading performance of CS, surface modification was carried out. CS (15 mg) was added to a 25 mL anhydrous ethanol and sonicated for 30 min to ensure thorough dispersion of CS. Then, added (3-aminopropyl) triethoxysilane (APTES, 0.15 mL) and stirred for 12 h at 60 °C. After that, the above reactant was cooled down to room temperature, washed three times with ethanol, and dried at 50 °C to obtain the surface modified CS. The surface modified CS (2 mg) and ICG (2 mg) were added into water (1 mL), and stirred for 24 h at 25 °C. Then, the mixture was washed by water and vacuum dried at room temperature. The ICG loaded CS nanoparticles (CSI) was acquired. Then, CSI (2 mg) and lysostaphin (2 mg) were added in water (1 mL) for incubation at 25 °C overnight. Finally, the lysostaphin loaded CSI (CSIL) was washed with water and vacuum dried at room temperature overnight, the CSIL nanomotor was then gained. The loading amounts of ICG and lysostaphin were determined by measuring the absorbance of the mixture solution before and after the loading process using a UV-vis spectrophotometer.

### Evaluation of the enzyme activity for lysostaphin

The activated MRSA bacterial suspension was diluted to an OD_620_ of 0.25-0.5. The group treated with lysostaphin alone served as the control, while the CSIL+NIR group was used as the experimental group. The reaction was conducted at 37 °C for 10 min, after which the OD_620_ value was measured again.

### Characterization of the motion behavior of CSIL

The motion trail of CSIL was observed under different laser irradiation power densities using an inverted fluorescence microscope and recorded in a video. The motion trajectory and mean velocity of CSIL in water were recorded by Video Spot Tracker software. Meanwhile, mean square displacement (MSD) was generated with the tool of MATLAB software.

### Evaluation of biofilm penetration ability of CSIL nanomotor

The transwell migration assay was employed to evaluate the ability of CSIL to penetrate the MRSA biofilm. In brief, MRSA (10^8^ CFU mL^-1^) was injected into the upper chamber. After the formation of biofilms, the dispersed solution of RhB-labeled CSIL in PBS (10 μg mL^-1^, 100 μL) was added to the upper chamber. Afterwards, under NIR irradiation, the absorbance of red fluorescence in the lower chamber was measured using a fluorescence microplate at varied time. Confocal laser scanning microscopy (CLSM) was also used to observe the characteristic of CSIL to penetrate the biofilm. Specifically, MRSA bacteria were implanted into a confocal 96 well plate to form a biofilm. After the formation of the biofilm, the dispersed solution of RhB-labeled CSIL in PBS (10 μg mL^-1^, 100 μL) was added to the plate and stained with STOY9 to produce green fluorescence. Afterwards, under NIR irradiation, the red fluorescence of the biofilm layer through confocal microscopy was recorded at varied time.

### Evaluation of the ability of CSIL against MRSA bacteria in vitro

MRSA bacterial was implanted in 48-well plate at 10^8^ CFU mL^-1^, the biofilm formed after 48 h. The biofilm was treated with PBS, CS, CSI, CSIL (200 μg mL^-1^, 500 μL) alone, or under NIR irradiation for 10 min (1.2 W cm^-2^). Subsequently, the obtained bacterial suspension was diluted step-by-step and coated on agar plates (100 μL), and incubated at 37 ℃ for 18 h. The bacterial survival rate was determined throughout counting the CFU on different plates. The bacterial survival rate was determined according to the following formula,

*C = B/A* × 100 %

where *C* indicates the percentage of survival, *A* represents the number of bacteria surviving in the control group, and *B* represents the number of bacteria surviving in each experimental group.

### RNA sequencing analysis of CSILs treated MRSA

MRSA (10^8^ CFU mL^-1^) was cultured, then treated with CSIL (200 μg mL^-1^) under NIR irradiation. Subsequently, the treated MRSA was collected and performed RNA sequencing analysis. For control group, normal MRSA was used. Logarithmically growing MRSA bacteria were extracted for RNA, and then reverse transcribed to measure QS, biofilm formation, and TCA-related gene expression by qPCR (Table S1).

### Infected wound healing experiments in diabetic mice

All protocols and ethnical rules for animal experiments were approved by Laboratory of Animal Center of the Xiangya School of Medicine, Central South University (Changsha, China). Continuously injected STZ (50 mg kg^-1^) into C57 mice *via* the peritoneal cavity for five days, ensuring 12 h fasting period before injection. One week later, the fasting blood glucose was measured, while the glucose greater than 11.1 mM selected as the diabetic mice. Shaved the dorsal region of the mice beforehand, and then anesthetized by tribromoethyl alcohol (240 mg kg^-1^). Next, the wounding with a diameter of approximately 8 mm was created on the back of mice and the MRSA bacterial was injected at the density of 10^8^ CFU mL^-1^ (10 μL). After two days, the MRSA biofilm developed, and the mice were divided into six groups, i*.e.*, PBS, CS, CSI+NIR, CSIL, CSIL+NIR and Van group. Three consecutive days of treatment was given on the back of surface at the dose of 5 mg kg^-1^, and the NIR groups were irradiated for 10 min (1.2 W cm^-2^). The wounds were photographed at day 0, 4, 8, 12, and 16, during the treatment period.

Until the end of experiment, the mice were euthanized and the wound skin was taken down. Placed the wound in a sterile PBS solution (2 mL) and used low-power ultrasound to disperse the suspension evenly, and incubated at 37 °C overnight. Gradually diluted the bacterial suspension and planted it onto agar plates, counted the bacteria after 18 h. Meanwhile, the wounding samples were fixed with 4% formaldehyde for subsequent histomorphometry analysis.

Hematoxylin-eosin (H&E) and Masson's staining were applied to analysis inflammation and collagen deposition in wound tissue. The immunohistochemical of CD31 and vascular endothelial growth factor (VEGF) were used to assess the angiogenesis. In addition, fluorescence staining for total macrophage markers (CD68), M1 macrophage markers (CD86) and M2 macrophage markers (CD206) was also performed. Besides, the immunohistochemical and enzyme linked immunosorbent assay (ELASA) were devoted to Interleukin-6 (IL-6) and Interleukin-10 (IL-10) and tumor necrosis factor-α (TNF-α) assessment. Moreover, blood analysis on fresh mouse blood and immunohistochemical analysis on organs covering heart, liver, spleen, lungs, and kidneys were performed to evaluate the biosecurity of CSIL nanomotors.

## Results and Discussion

### Preparation and characterization of nanomotors

The design, preparation and applications of nanomotors is depicted in **Scheme 1**. Firstly, the nanomotors were prepared according to the previous reporeted procedures with a slight modification 31. Briefly, a nanosphere of RFR/SiO_2_@RFR with a resorcinol formaldehyde resin (RFR) core and a shell of RFR and SiO_2_ mixture was prepared. Then, Al(NO_3_)_3_ was used to etch RFR from the RFR/SiO_2_@RFR nanospheres. Resorcinol formaldehyde resin was partially etched in both the core and the shell of the nanospheres. After etching, the nanospheres were placed in a tube furnace for calcination, and RFR in the nanospheres was carbonized, resulting in the formation of eccentric yolk-shell structured C/SiO_2_@C (CS) nanoparticles with spinous surfaces. To improve the loading performance of CS, (3-aminopropyl) triethoxysilane (APTES) was grafted to its surface to make it positively charged. Finally, the surface modified CS was used as a carrier to load ICG (named CSI nanomotor) or co-load ICG and lysostaphin (named CSIL nanomotor).

**Figure 1:**
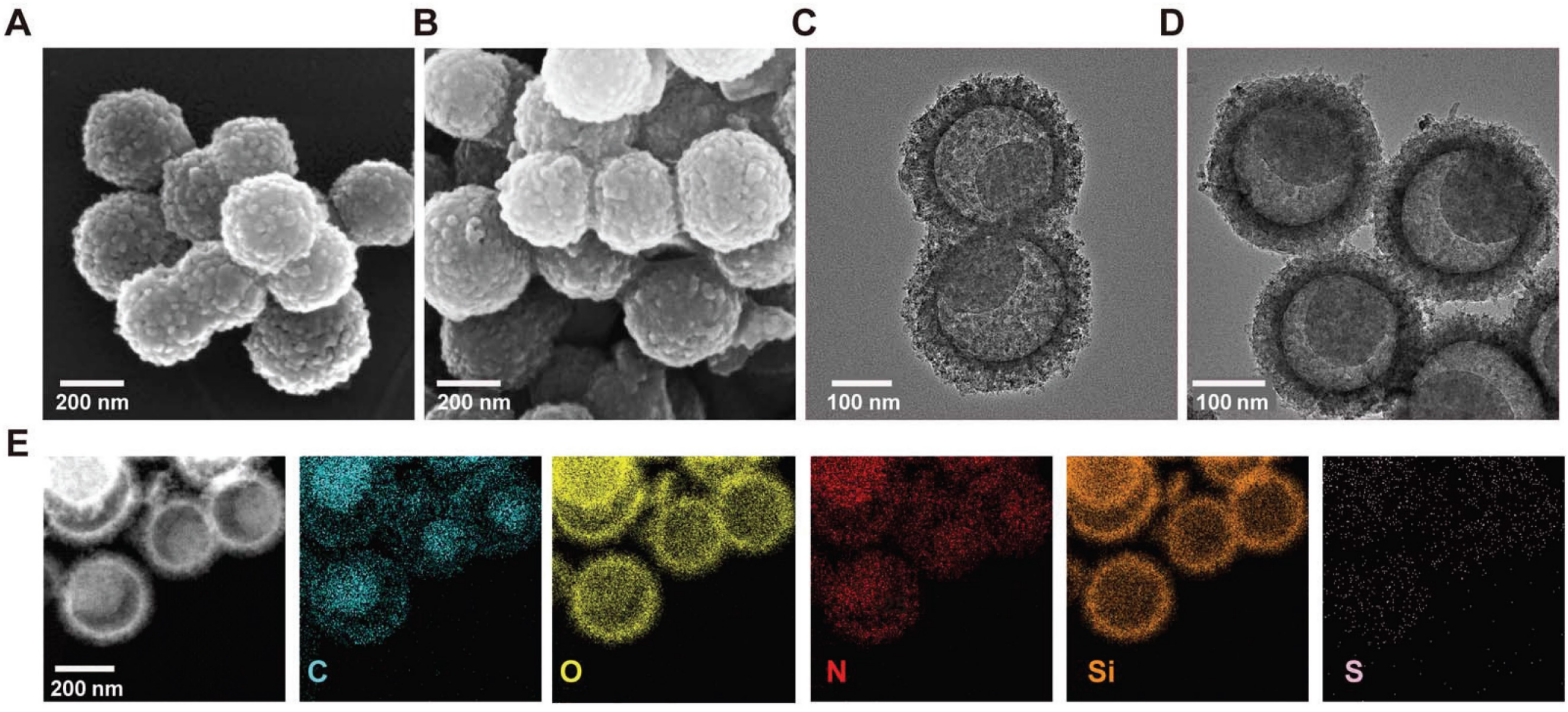
(A) Scanning electron microscopy image (SEM) of CS nanomotor. (B) SEM image of CSIL nanomotor. (C) Transmission electron microscopy (TEM) image of CS nanomotor. (D) TEM image of CSIL nanomotro. (E) TEM image and corresponding element mapping of CSIL nanomotor.

Scanning electron microscopy (SEM) and transmission electron microscopy (TEM) were used to visualize the surface and the morphologies of CS and CSIL. As shown in **Figure 1A**-**B**, both CS and CSIL nanomotors possess yolk-shell structures with diameters of about 250 nm. The TEM images clearly reveal that CS and CSIL are of eccentric carbon yolk structure. In addition, after loading ICG and lysostaphin, although the surface of CSIL became rougher, its structure remains unchanged compared to CS (see **Figure 1C**-**D**). As shown in **Figure 1E**, it can be clearly observed that C and Si elements are uniformly distributed in the core and shell. Besides, the S and N elements, which contains in APTES, ICG and lysostaphin, appear in both the inner and outer shell, indicating that ICG and lysostaphin are successfully loaded on CS. The eccentric yolk-shell structure of the CSIL nanomotor exhibits a distinct geometric asymmetry. Under NIR light irradiations, the nanomotor with a carbon-core structure demonstrates a high photothermal conversion efficiency, while the SiO_2_ shell absorbs less heat. Due to this temperature gradient, the nanomotor experiences asymmetric molecular thermal motion between the high-temperature and low-temperature regions, propelling it to move along the direction of the temperature gradient. UV-vis spectroscopy was performed to measure the loading efficiency. As shown in **Figure S1A**-**B**, the characteristic absorption peaks of ICG and lysostaphin are located at 800 and 280 nm, respectively. The loading efficiencies of ICG and lysostaphin are calculated to be 52% and 82%, respectively (**Figure S1C**). Moreover, the zeta potential of the modified CS is 10.33 mV, and after loading with negatively charged ICG, the potential of CSI changes to -6.93 mV. After further loading with positively charged lysostaphin, the potential of CSIL significantly increases to 11.72 mV, further confirming the successful loading of ICG and lysostaphin on CS (**Figure S1D**).

### Photothermal, penetrability and targeting ability of nanomotors

**Figure 2:**
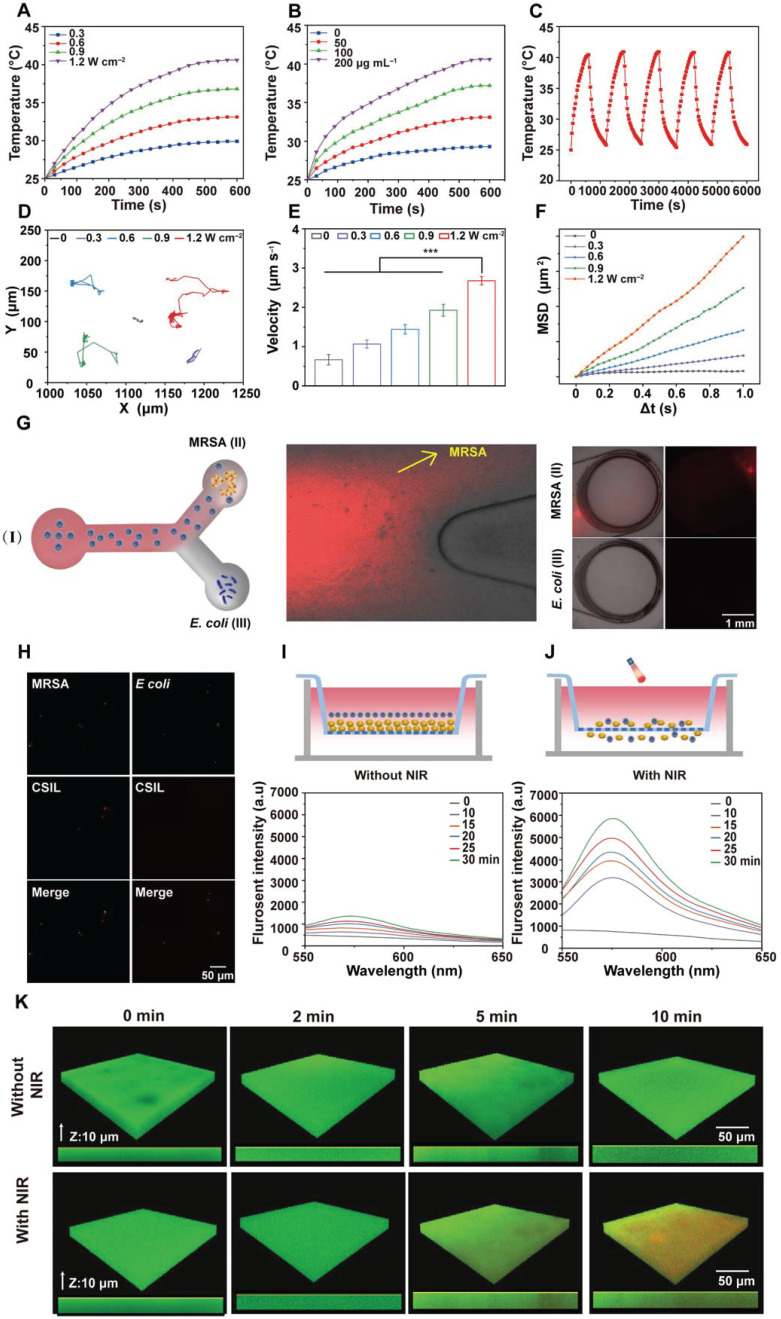
Evaluation of the photothermal and movement performance of nanomotors. (A) The variation in temperature rise with time on CSIL (200 μg mL^-1^) under NIR irradiaton with different power densities. (B) The variation in temperature rise with time under NIR irradiaton (1.2 W cm^-2^) on different concentrations of CSIL. (C) Recycling heating fluctuation of CSIL (200 μg mL^-1^) under NIR irradiation (1.2 W cm^-2^) for five on/off cycles. (D) Trajectories of CSIL under varied NIR power densities. (E) Average velocity of CSIL under NIR with various power densities, ***p < 0.001. (F) The mean square displacement (MSD) of CSIL under NIR with different power densities. (G) Sketch diagram of the Y-shaped channel of CSIL, reservoir (I) containing RhB-dyed CSIL, reservoir (II) containing MRSA in reservoir (III) containing *E*. coli; red fluorescent images of the reservoir (II) and (III) at 30 min. Scale bar: 1 mm. (H) Fluorescent images of nanomotors bound to MRSA and *E. coli* at 30 min respectively (green: bacteria; red: CSIL). (I) Fluorescence spectra of the RhB-dyed CSIL in the low chambers without NIR irradiation. (J) Fluorescence spectra of the RhB-dyed CSIL in the low chambers with NIR, (upper chamber inoculated with MRSA). (K) Penetration depth of CSIL nanomotor in MRSA biofilm under NIR propulsion.

The photothermal performance of nanomotors under 808 nm laser irradiation at different power densities was evaluated. As shown in **Figure 2A** and **Figure** S**2A**-**C**, when NIR irradiation for 10 min with 1.2 W cm^-2^, the temperature of CS and CSI increases from 25.0 to 31.7 and 41.2 °C , respectively. Especially, when CSI was loaded with lysostaphin, its temperature rises to 40.3 °C , indicating that the loading lysostaphin has little effect on the photothermal performance of CSIL. The photothermal performance of CSIL also present a concentration-dependent behavior (**Figure 2B**). Moreover, as illustrated in **Figure 2C** and **Figure S2D**-**E,** CS, CSI and CSIL nanomotors exhibit high photothermal stability and recyclability. The photothermal conversion efficiency (*η*) of CSIL was calculated to be 46.6% (**Figure S2F**). Besides, to investigate the effect of ICG on Ly enzyme activity, we tested the enzyme activity of Ly alone and Ly in CSIL. As shown in **Figure S3A**, compared to using Ly alone, the activity of Ly in CSIL only displays slightly decreased, indicating that the photothermal effect of ICG on enzyme activity can be ignored.

The motion trajectory of CSIL nanomotor under NIR irradiation was recorded by inverted fluorescence microscopy. As displayed in **Movie S1**-**5**, without NIR irradiation, CSIL exhibits limited and irregular Brownian motion. In contrast, with NIR irradiation, the diffusion range of nanomotors gradually expands and their motion trajectory significantly extends (**Figure 2D**). Moreover, the average speed of CSIL reaches 2.67 μm s^-1^ after irradiation at 1.2 W cm^-2^, representing four times quicker than that of the control group (**Figure 2E**). Finally, the mean square displacement (MSD) of CSIL nanomotor was calculated using MATLAB software 4. As shown in **Figure 2F,** the diffusion speed increases with the increase of irradiation power.

To validate the precise targeting of MRSA bacteria and effective penetration of biofilm by CSIL, Y-tube experiments were conducted. As shown in **Figure 2G**, a solution of CSIL containing Rhodamine B (RhB) dye was introduced into the inlet of a Y-tube (I), with pre-cultured MRSA (II) and *E*. coli (III) at the ends of the Y-tube separately. Upon light-driven propulsion of CSIL in (I), it can be observed that the fluorescence intensity towards MRSA at the bifurcation of the Y-tube is significantly higher than that towards *E*. *coli*, with greater accumulation in (II) compared to (III) over time. In addition, the tight binding between CSIL (red fluorescence) and MRSA (green fluorescence) is observed by confocal microscope, while only a minimal overlap between *E*. coli (green fluorescence) and CSIL (**Figure 2H**) appears. These results confirm the specific targeting ability of CSIL to MRSA through NIR propulsion. It is noted that a minor proportion of motors diverted towards *E*. *coil* at the fork of the Y-tube, which may be attributed to motion inertia.

Meanwhile, the penetration performance of CSIL to MRSA biofilm was investigated using Tramswell experiment. As illustrated in **Figure 2I**-**J**, upper compartments infected with MRSA for 48 h formed thick golden biofilms. Subsequently, RhB-dyed CSIL was applied into this space as well. Without NIR irradiation, only a small quantity of CSIL entered the lower compartment due to gravity as indicated by a slight increase in fluorescence intensity. In contrast, after NIR irradiation, CSIL penetrated through the biofilm and aggregated in the lower chamber, leading to rapid rise in fluorescence intensity over time. In addition, compared with the non-irradiated CSIL group, the fluorescence intensity of RhB in the CSIL group increases by 5.15 times after NIR irradiation for 30 min (**Figure S3B**), which confirms the superior deep-penetration capability of CSIL. Similarly, confocal laser scanning microscopy (CLSM) analysis shows that the red fluorescence of CSIL invading the biological membrane from top to bottom, further demonstrating the powerful propelling force of NIR for nanomotor (**Figure 2K**). Therefore, CSIL nanomotor propelled by NIR exhibits high targeting activity towards MRSA and has a strong ability to penetrate MRSA biofilms, providing a solid foundation for subsequent biological experiments.

ICG is a near-infrared fluorescent dye that can absorb light to convert it into heat and generate ^1^O_2_, and has been applied in photodynamic and photothermal therapy. DPBF probe was used to detect the generated ^1^O_2_. As shown in **Figure S4,** when CSI and CSIL were exposed to 808 nm laser irradiation, the typical absorption peak of DPBF at 410 nm gradually weakened with time, indicating efficient production of ^1^O_2_. Moreover, under NIR irradiation, the ability of CSIL to produce ^1^O_2_ remains unchanged compared to CSI, indicating that the loading lysostaphin has almost no effect on the photodynamic performance of ICG **(**see **Figure S5).**

### *In vitro* anti-biofilm activity of CSIL

**Figure 3:**
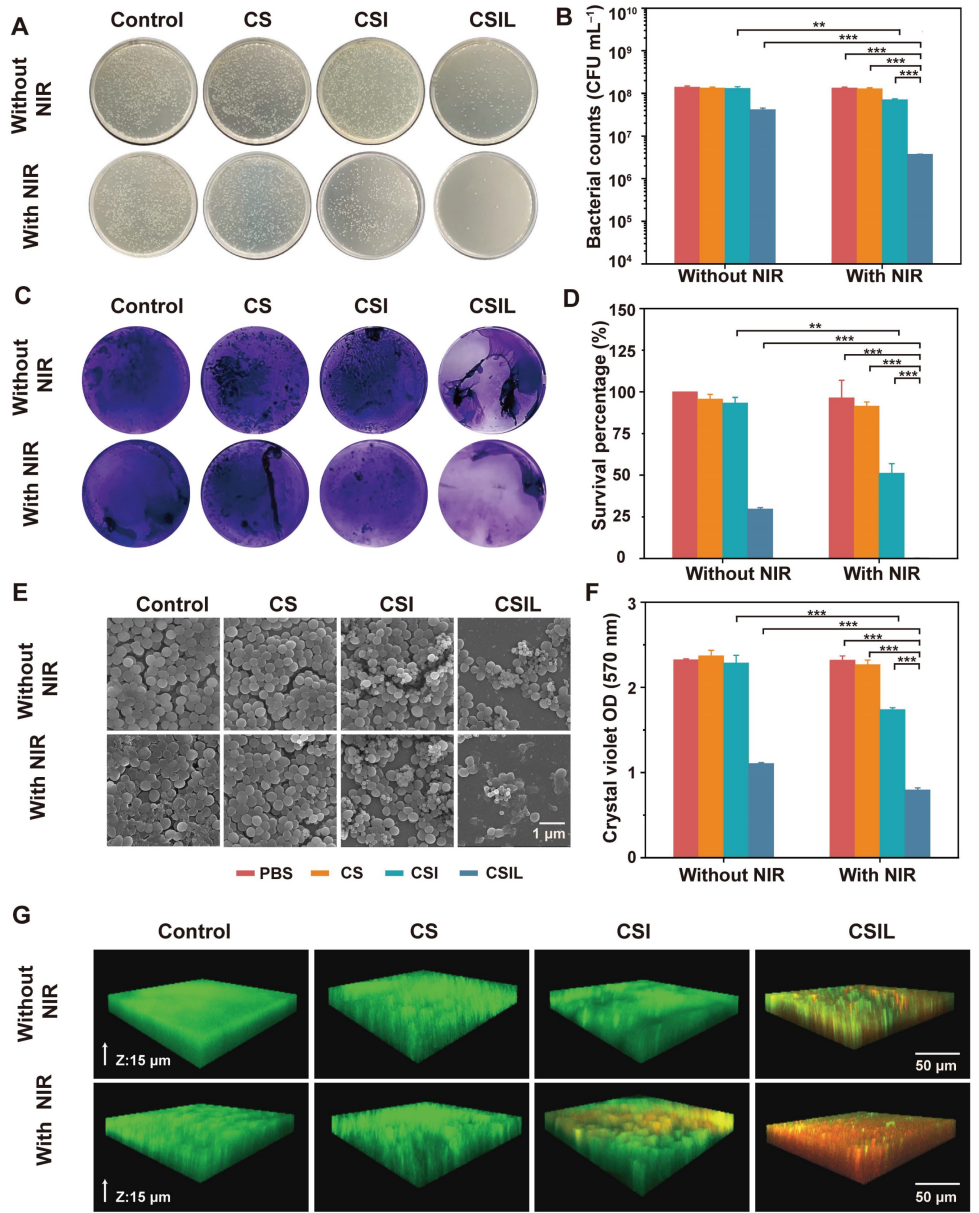
Antibiofilm properties of CSILs *in vitro*. (A) Representative plates of bacterial colonies isolated from MRSA biofilms after different treatments. (B) The amount of MRSA after different treatments (PBS, CS, CSI, CSIL with or without NIR), *p < 0.05, **p < 0.01, ***p < 0.001. (C) Crystal violet staining of MRSA after varied treatments. (D) Survival percentage of MRSA after different treatments. (E) SEM images of MRSA after different treatments. (F) Crystal violet optical density (OD) of MRSA. (G) Live/dead staining images of MRSA and biofilms under CLSM observation, where the green fluorescence represents live bacterial, while the red fluorescence stands for dead bacterial.

Biofilms are defined as rigid structures composed of bacterial communities and extracellular polymeric substances, which possesses physical stability against mechanical removal and act as a barrier to antibacterial diffusion in the way of electrostatic adsorption and enzymatic degradation. Therefore, the elimination of biofilm is the top priority of antimicrobial therapy 34-36. Firstly, *in vitro* experiments were conducted to treat MRSA biofilms with nanomotors under NIR irradiation. As shown in **Figure 3A,** the agar plate counting results reveal that there are a large number of colonies in the groups without NIR irradiation, except for the CSIL group. In contrast, under NIR irradiation, the number of MRSA colony forming units (CFU) in the CSI group decreases by an order of magnitude, compared to those of the PBS and CS groups. These results also indicate that the effect of NIR light irradiation on CS nanomotor is not as significant as that of CSI, which suggests that the ^1^O_2_ generated by loading ICG can effectively improve the ability of elimination MRSA biofilm. In addition, it is worth noting that when treated with CSIL+NIR, MRSA colonies decrease by three orders of magnitude (equivalent to a 99.7% reduction in bacterial survival rate), indicating its good bactericidal performance (see **Figure 3B** and **D**). Interestingly, CSIL shows a bactericidal rate of about 70% without the use of NIR irradiation, further verifying the strong targeting effect of lysostaphin to MRSA. As depicted in **Figure 3C** and **F**, when treated with CSIL+NIR, the crystal violet stained biofilms dissipate to a large extent, with only a few purple biofilms present and a significant decrease in absorbance intensity (570 nm).

In addition, SEM was used to observe the morphology alterations of MRSA. As shown in **Figure 3E**, the spherical bacteria exhibit a complete shape with an intact biofilm. When treated with CSI+ NIR, some dead bacterial cells gradually dissipate the biofilm attribute to the bactericidal effect of ^1^O_2._ Obviously, due to the antibacterial ability of lysostaphin, the dead bacteria are widely extensively distributed in the CSIL+NIR group, exhibiting distorted or fragmented morphology, which more intuitively demonstrates the efficacy of CSIL in eradicating persistent MRSA biofilms and killing bacteria *in vitro*.

Moreover, live/dead cell staining experiments were used to evaluate the antibiofilm effect of the nanomotors. Green fluorescence represents live bacteria, while red fluorescence represents dead bacteria. Although under NIR irradiation, the PBS and CS groups show consistent green fluorescence throughout the biofilm layer, with little red fluorescence, confirming the compactness of biofilms. After NIR irradiation, superficial red fluorescence appears in the CSI group, suggesting partial eradication of the biofilms. In contrast, in CSIL group, red fluorescence appears throughout the entire biofilms, indicating complete dissolution of the biofilm and many bacterial deaths (see **Figure 3G**). Therefore, CSIL possesses remarkable penetration ability against MRSA biofilms *in vitro*.

Furthermore, CSIL nanomotor was applied to HUVEC and NIH-3T3 fibroblasts for cell viability assays. As shown in **Figure S6**-**8**, even at concentration of CSIL up to 400 μg mL^-1^, the cell survival rates of HUVEC and NIH-3T3 remain above 90%, indicating that CSIL nanomotor has good targeting properties for biofilms. Furthermore, to evaluate the biocompatibility of the CSIL nanomotor, CSIL was incubated in red blood cell suspension (2%) using PBS as the negative control and deionized water as the positive control. As shown in **Figure S9**, even when the concentration of CSIL reaches 400 μg mL^-1^, the hemolysis rate is only 4.8%, indicating excellent blood compatibility of CSIL.

### Regulation of MRSA transcriptome by nanomotors

**Figure 4:**
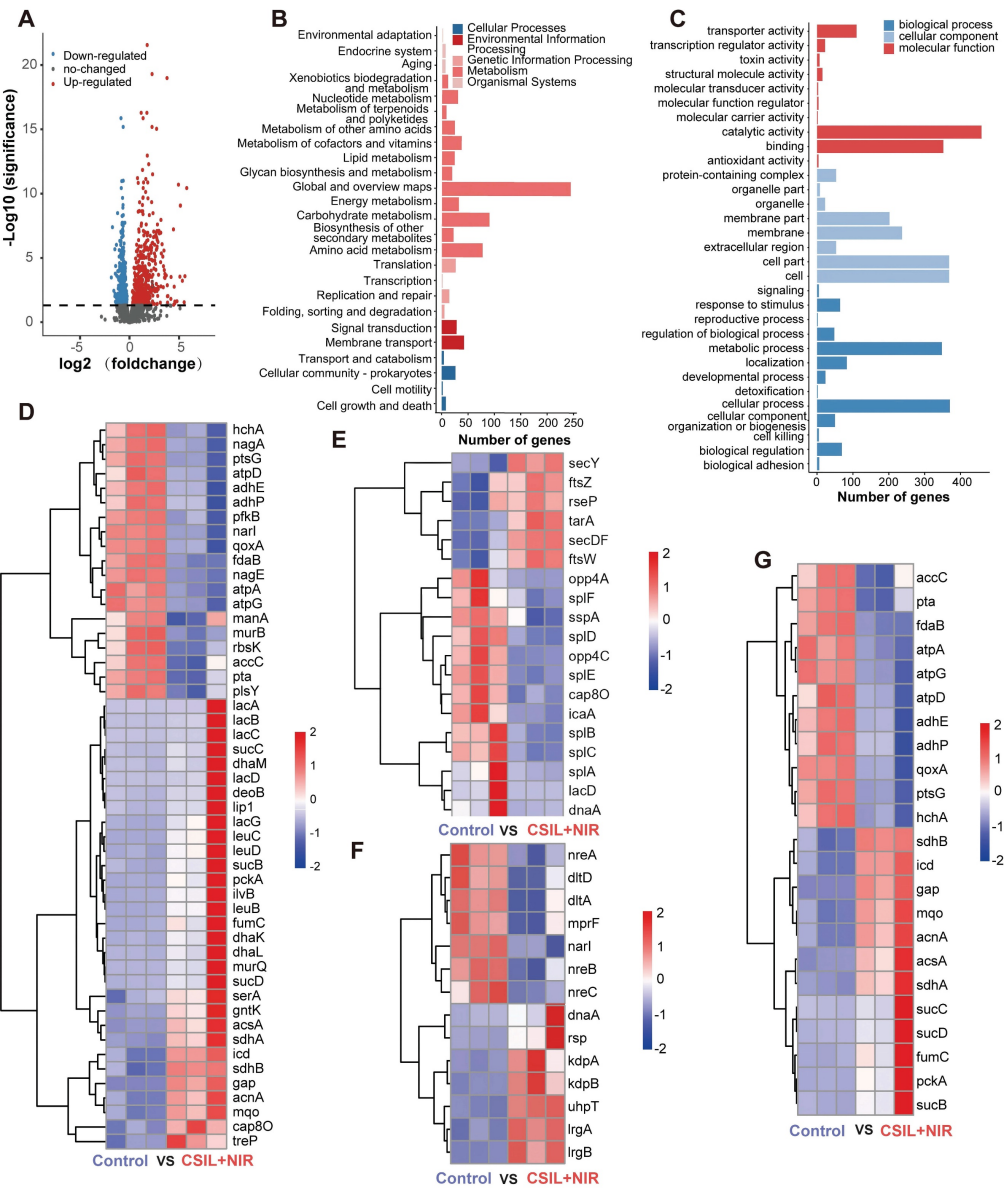
Regulation of the MRSA transcriptome by CSIL nanomotor. (A) Volcano plot analyses of total DEGs in MRSA after treatment with PBS and CSIL+NIR. Red and blue dots represent upregulated and downregulated genes, respectively. Gray dots represent the genes without significant difference. (B) KEGG analysis of DEGs. (C) GO analysis of DEGs. (D) Heat map of genes associated with energy metabolism, carbohydrate and lipid metabolism. (E) Heat map of genes associated with biofilm, QS and cell growth and death. (F) Heat map of genes associated with the two-component signal transduction system (TCS). (G) Heat map of genes associated with TCA, oxidative, phosphorylation and pyruvate metabolism.

To get an insight into the mechanism of antibiofilm of CSIL under NIR irradiation, the regulation of MRSA transcriptome by CSIL nanomotor was investigated by transcriptome sequencing (RNA-seq). As shown in volcano plot, 842 different expressed genes (DEGs) were extracted from the normal MRSA and MRSA treated with CSIL. Among them, there are 468 upregulated genes and 374 downregulated genes (**Figure 4A**). Next, an analysis was conducted on the Kyoto Encyclopedia of Genes (KEGG) and Gene Ontology (GO) pathway. It is found that there are 365 and 742 DEGs are enrich in KEGG and GO terms respectively, involving various metabolic and biological processes (**Figure 4B**-**C**). The maintenance of bacterial metabolic homeostasis is the foundation of their survival. Previous studies have shown that after antibiotic treatment of MRSA, the metabolic activity of the bacteria is augmented under stressful conditions 37. The sequencing results reveal that the genes related to carbohydrate metabolism, including deoB, sdhA and lac genes, are upregulated in CSIL+NIR group. Similarly, energy metabolism related genes, such as acnA, gap, pckA and sucC are also elevated after treated by CSIL+NIR. However, it is worth noting that certain genes in the CSIL+NIR group are downregulated, which may be due to the production of toxins during the bacterial death, thereby hindering their metabolic processes. These results indicate that the treatment of CSIL+NIR caused stress response in MRSA (**Figure 4D**).

QS is a communication system that essential to many physiological processes in bacteria, including biofilm formation, toxin production, antibiotic resistance and etc.^38^, ^39^. Hemolysin is an essential component in QS, which can induce red blood cell lysis and produce toxins to the host 40. It is reported that the phospholipase encoded by the splA gene is able to destroy host cells tgrough out the cooperation with MRSA α-hemolysin 41. A significant downregulation in splA gene expression in the CSIL+NIR group is observed. In addition, the heatmap also shows significant downregulation of other QS related genes, including opp4C, lacD, and sspA, which are mainly involved in virulence regulation, tissue invasion, and immune evasion 42-44. Moreover, compared with the control group, the biofilm core gene icaA and cap8O that responsible for synthesis of polysaccharide intercellular are downregulated (**Figure 4E** and **Figure S10A**-**D**) 45, 46. In this case, the tight adhesion between bacteria is easily disrupted, further confirming the bactericidal and biofilm breaking effects of CSIL+NIR. Generally, bacteria activate the two-component signal transduction system (TCS) to maintain their reproduction and virulence to adapt to adverse environments 47. As expected, in the treatment group, LrgA and LrgB genes regulating bacterial programmed cell death were upregulated, while genes related to toxicity such as dltD were down regulated (**Figure 4F** and **Figure S10E**) 48, 49. During the tricarboxylic acid cycle (TCA), most TCA related genes were upregulated in the CSIL+NIR group. However, gene components involved in oxidative phosphorylation and glycolysis, such as ATP synthase (atpA), cytochrome oxidase subunit (qoxA), and phosphotransferase (ptsG), show a decreasing trend, indicating that respiratory chain and ATP production were hindered (**Figure 4G** and **Figure S10F**) 50-52. In summary, the CSIL+NIR treatment can inhibit bacterial QS system, disrupt biofilm structures, and hinder bacterial reproduction.

### Anti-biofilm activity of CSIL in diabetic wounding mice

**Figure 5:**
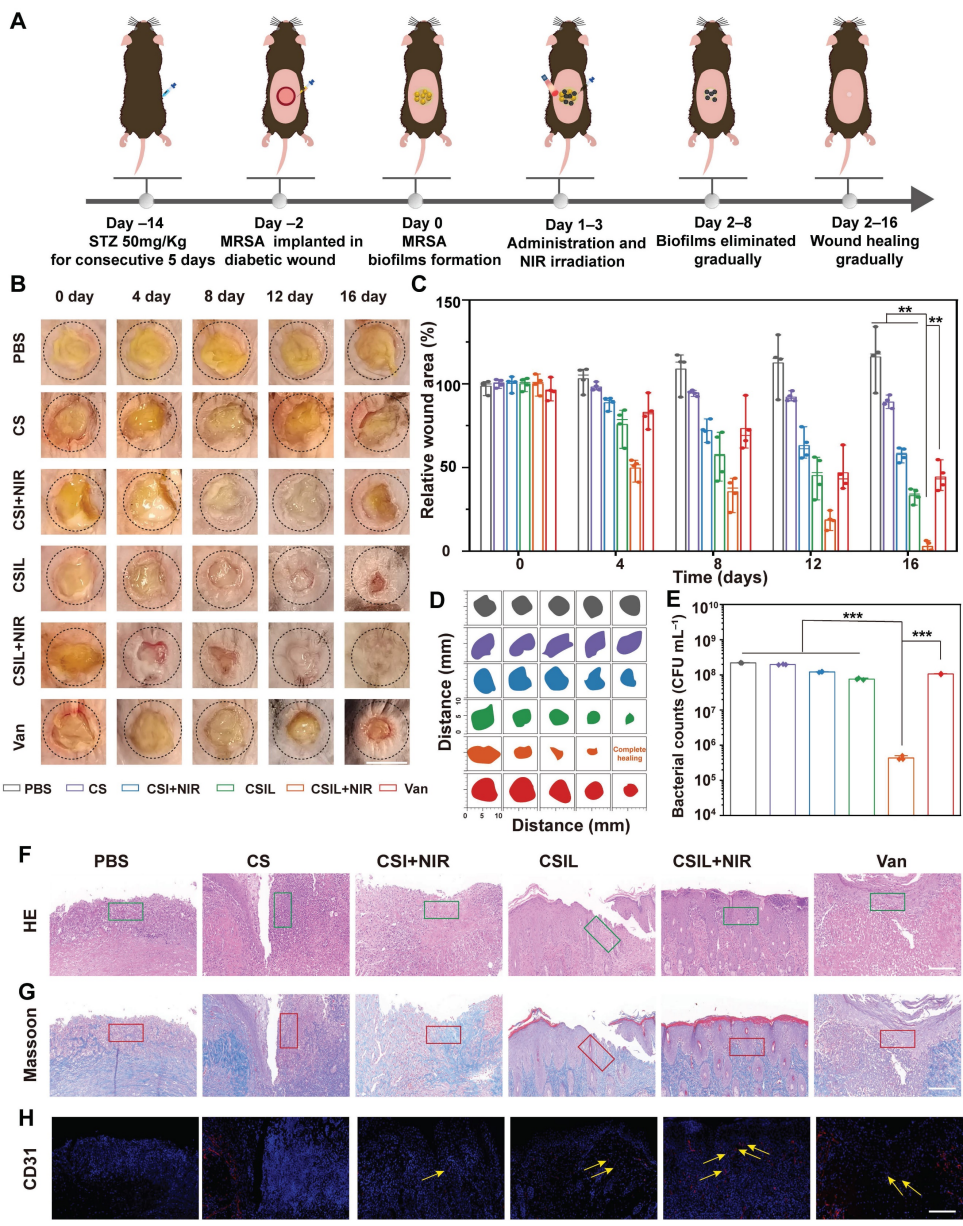
Evaluation of the wound treatment effects of CSILs in MRSA-infected diabetic mice *in vivo*. (A) Diagram of diabetic wound modeling and treatment process. (B) Photographs of wounds in 0, 4, 8,12, 16 days respectively after treatment with PBS, CS, CSI+NIR, CSIL, CSIL+NIR and Van. Scale bar: 8 mm. (C) Percent wound area change in different treatment groups. (D) Traces of wound closure over a 16 days different treatment. (E) Bacteria number of wounding in different groups after a 16 days period of treatment, ***p < 0.001. (F-H) Results of H&E, Masson's, and CD31 staining of injured skin in different treatment groups after a 16 days treatment. Green boxes represent inflammatory infiltration area; the red box represents collagen deposition; yellow arrows represent neovascularization, Scale bar:100 μm.

Encouraged by the outstanding performance of CSIL in eliminating MRSA biofilms *in vitro*. We further extended its application to the treatment of diabetes wound models implanted with MRSA. As illustrated in **Figure 5A**, C57 mice were intraperitoneally injected with STZ for 5 consecutive days. One week later, a standard of the fasting blood glucose level of diabetes mice was more than 11.1 mM. Subsequently, a wound with a diameter of 8 mm was created on the dorsal region of the mice, and MRSA was inculated at a concentration of 10^8^ CFU mL^-1^. After 48 h, biofilm formation can be observed. Then, MRSA infected wounds were treated with PBS, CS, CSI+NIR, CSIL, CSIL+NIR, and vancomycin (Van), respectively. Each mouse in the NIR group was exposed to NIR light for about 10 min. Infrared photothermal images of different groups of mice show that the temperatures of the PBS group and CS group increase to 27 and 32.6 °C , respectively. While the temperature of the CSI group and CSIL group reaches 41.7 and 40.8 °C , respectively (**Figure S11**).

Under the temperature sensitive condition below 45 °C , CSIL nanomotor can release ^1^O_2_ at the initial treatment. When the light off, after loading with Ly, CSIL motor penetrate the biofilm to release ^1^O_2_, producing synergistic antibacterial effect, which can also protect diabetes wounds from secondary damage caused by hyperthermia. Apparent to the naked eye, the wound treated with CSIL + NIR exhibits a significant removal in biofilm on the day 4, whereas persistent biofilm is observed in both the PBS group and CS group. Furthermore, compared to the wound area of PBS group (116%) by day 16, the wound has almost complete healed in CSIL +NIR group (2.8%), and improved in CSI + NIR (57.8%), CSIL (33.2%) groups (**Figure 5B**-**D**). After termination of different treatments, the remnant bacteria in the wound were counted, which shows that compared with the PBS group, the bacterial count in CSIL+NIR decreases by 3.0 log10, and the antibacterial rate reaches 99% (**Figure 5E** and **Figure S12**).

However, there is no order of magnitude decrease in CSI+NIR and CSIL groups, with bacteriostasis rate of around 45% and 66%, respectively. Even with the front-line drug vancomycin used to treat MRSA infections, the bacterial reduction shows only 1.0 log10, which may be attributed to surface administration (**Figure S13**). It is reported that the efficacy of oral vancomycin was minimal in patients with severe MRSA pneumonia and burn injuries 53. Nevertheless, intravenous injection requires careful monitoring of drug concentration and renal function, which limits its bactericidal potential 54. Therefore, compared to Van, CSIL nanomotor can combat MRSA biofilms and ensure biosafety.

Skin healing requires sever synergistic steps including inflammatory resolution, collagen deposition, and angiogenesis. As shown by H&E staining, in the PBS and CS groups, a large number of inflammatory cell infiltrates are recruited as "first responders" for antibacterial treatment (green border) 55. Instead, there were few inflammatory cells in the CSIL group, and the skin structures are completely restored (**Figure 5F**). Consistently, the inflammatory infiltration area of CSIL+NIR was calculated to be 4.21%, which is lower than the areas of PBS (56.1%), CS (51.9%), CSI+NIR (40.8%), CSIL (24%) and Van (31%) (**Figure S14**).

The orderly arrangement of collagen can promote wound closure and enhance the proliferation of extracellular matrix cells, thereby resulting in tissue remodeling. Obviously, a large amount of well-organized collagen deposition is observed in the CSIL+NIR group, accounting for approximately 74% of the total area (red border), while collagen deposition in PBS, CS, CSI+NIR, and Van only account for 14.7%, 16.3%, 32.4%, and 31.5%, respectively (**Figure 5G** and **Figure S15**). Neovascularization is crucial for rebuilding tissue nutrition and maintaining cellular homeostasis 56. As expected, significantly enriched germ tubes (yellow arrows) were observed in the CSIL+NIR group, accompanied by 14.4% CD31 density, significantly higher than 2% in PBS and 2.6% in CS, as well as 5.7% in CSI, 7.5% in CSIL, and 4.8% in Van (**Figure 5H** and **Figure S16**). It is worth noting that CSIL exhibits high efficacy in eradicating MRSA biofilm and diabetes wound regeneration *in vivo* experiments, even superior to vancomycin, demonstrating great potential for clinical application.

**Figure 6:**
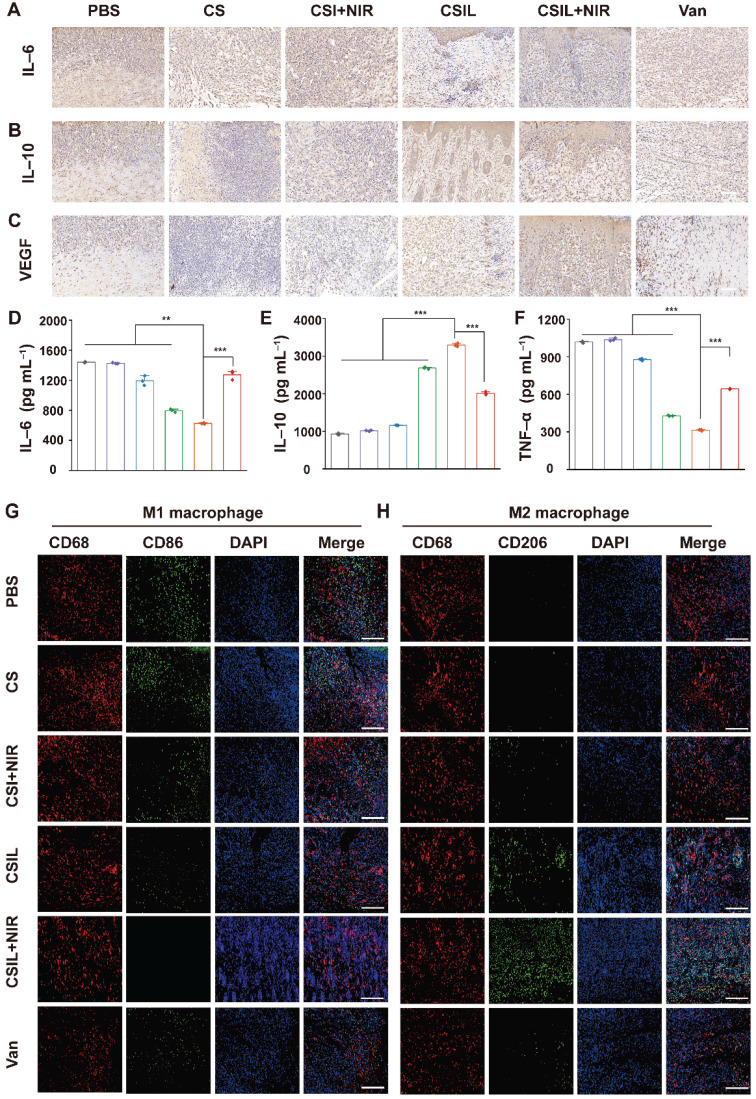
Immunohistochemical analysis of sections. (A-C) Immunohistochemical staining of IL6, IL10 and VEGF in different treatment group, respectively. Scale bar: 100 µm. (D-F) The serum level of IL6, IL10 and TNF α detected by ELASA in different treatment group, respectively. (G) Immunofluorescence staining of macrophage phenotype markers in wound tissues: Total CD68 macrophage (red) and CD86 M1 macrophage (green). (H) Immunofluorescence staining of macrophage phenotype markers in wound tissues: CD68 pan macrophage (red) and CD206 M2 macrophage (green). Scale bar: 100 µm.

Generally, macrophages are crucial for wound healing and tissue remodeling. In the initial stage of wound healing, M1 mainly plays a role in bacterial killing and promoting inflammation. However, the impairment of M1 function in diabetes patients leads to delayed removal of neutrophils and ECM debris, which results in persistent chronic inflammation. In addition, inhibiting the transformation of M1 to M2 cells hinder angiogenesis and matrix remodeling, leading to delayed wound healing 57, 58. IL-6 is a pro-inflammatory cytokine originating from M1. Immunohistochemical quantitative analysis reveals that the positive cumulative area of IL-6 in the CSIL+NIR group is 7.9%, which is much lower than that in the PBS (30.6%), CS (29.1%), CSI+NIR (21.1%), CSIL (14.3%), and Van (17.2%) groups (see **Figure 6A** and **Figure S17)**.

On the contrary, IL-10 secreted by M2 is abundant in CSIL+NIR group, with a positive proportion of 42.8%, much higher than those in other groups (**Figure 6B** and **Figure** S**18)**. Meanwhile, the expression level of vascular endothelial growth factor (VEGF) was evaluated. As shown in **Figure 6C** and **Figure S19**, the expression of VEGF in CSIL+NIR group is the highest (45.8%), much higher than those in the PBS group (14.3%), CSIL group (32.1%), and Van group (20.9%), indicating good vascular regeneration ability. Similarly, compared with the CSIL+NIR group, the levels of IL-6 and TNF-α detected by ELASA are significantly increased in PBS, CS, CSI+NIR, CSIL, and Vans groups, while the levels of IL10 is opposite to that of IL6 and TNF-α (**Figure 6D**-**F**). Furthermore, the immunofluorescence was employed to label the surface markers of M1 and M2. The results indicate that CD206 (M2) is widely distributed in CSIL+NIR group, while CD86 (M1) is almost absent. On the contrary, other groups with poor wound healing show the presence of M1 cells (**Figure 6G**-**H**).

It is worth noting that during the first three days of NIR treatment, a large amount of ROS is generated, which works synergistically with Ly to eliminate the biofilms on the surface and shallow layers of the wound. During this period, the wound is in a pro-inflammatory state, characterized by the abundant presence of M1 macrophages. As we know, ROS is a double-edged sword, to reduce the side effects of ROS on wound healing, after 3 days of NIR treatment, the irradiation was no longer used. It can be observed that without the use of NIR, although ROS gradually decreases, CSIL can still continue to penetrate the biofilm through the targeting effect of Ly, then decomposed MRSA in deep layers. In addition, Ly not only inhibits pro-inflammatory cytokines, but also has antioxidant effect, which promotes wound 59. Therefore, as the treatment time prolongs, the wound tissue becomes dominated by M2 macrophages, promoting tissue repair and healing. Thus, CSIL nanomotors can alleviate inflammation, promote angiogenesis, and accelerate wound healing by regulating macrophage phenotype.

To evaluate the biosafety of CSIL nanomotor *in vivo*, tests were conducted on mouse body weight, liver and kidney function indicators, blood cell count, and H&E staining of major organs. The weight fluctuations of mice in different treatment groups are depicted in **Figure S20**. It can be observed that during the treatment period, the body weights of the mice in each group remains basically unchanged, indicating that the effect of CSIL nanomotor on mouse body weight can be ignored. In addition, compared with the control group, there is no statistically significant difference in blood biochemical indicators, such as kidney and liver indicators (**Figure S21**). The H&E staining maps of the heart, liver, spleen, lung and kidney reveal no abnormalities in any of the major organs (**Figure S22**). Therefore, CSIL nanomotor has good biosafety, and has great application potential in the treatment of chronic diabetic foot ulcers.

## Conclusion

In summary, a new type of nanomotor targeting MRSA infection has been designed. This nanomotor integrates three main components, *i.e.,* yolk-shell C/SiO_2_@C with eccentric structure, ICG, and lysostaphin.

The photothermal effect of eccentric yolk-shell C/SiO_2_@C drives the self-propulsion of nanomotors; ICG is a photosensitizer that has photothermal and photodynamic effects; lysostaphin has the ability to target MRSA biofilms and resist bacteria. At the beginning of the treatment, CSIL nanomotor can permeate into the biofilm under the irradiation of NIR, accompany with the ROS generated by ICG and lysostaphin-mediated cell membrane hydrolysis, achieving a synergistic bactericidal effects ultimately. Then, without applying NIR light radiation, the nanomotor can continuously penetrate biofilms under the targeted action of lysostaphin, achieving the eradication of deep biofilms. In the diabetic trauma model implanted with MRSA, during the process of biofilm banishment, a reduction in inflammation occurs, followed by collagen deposition and subsequent neovascularization, leading to desirable wound healing. Moreover, CSIL nanomotor modulates macrophage immunophenotype from M1 to M2 state which facilitates the transition from inflammatory to remodeling stage. This work reveals the the NIR light-driven nanomotor using cascade photodynamic therapy (CPDT) strategy can eradicate MRSA biofilm and promote wound healing, which provides new insights for the management of diabetes foot ulcers infected with MRSA.

## Supplementary Material

Supplementary materials and methods, figures and table.

Supplementary video: motion of CSIL, control AVI.

Supplementary video: motion of CSIL at 0.3 W-AVI.

Supplementary video: motion of CSIL at 0.6 W-AVI.

Supplementary video: motion of CSIL at 0.9 W-AVI.

Supplementary video: motion of CSIL at 1.2 W-AVI.
