# Supplementary material for: NIR light-driven nanomotor with cascade photodynamic therapy for MRSA biofilm eradication and diabetic wound healing
Source: Theranostics. 2025 Feb 24;15(8):3474–89. doi: 10.7150/thno.109356 (PMC11905136; doi:10.7150/thno.109356)
Supplement: Supplementary file 1 — Supplementary materials and methods, figures and table. [file thnov15p3474s1.pdf]

# Supplementary Data

**NIR light-driven nanomotor with cascade photodynamic therapy for MRSA biofilm eradication and diabetic wound healing**

Yuanyuan Deng[1], Jia Zheng[2], Jianghua Li[2], Bo Liu[1], Ke Chen[1], Yiling Xu[1], Liu Deng[2,*], Huixia Liu[1,*], and You-Nian Liu[2,*]

[1] Department of Geriatric Endocrine, Xiangya Hospital, Central South University, Changsha, Hunan 410083, China

[2] College of Chemistry and Chemical Engineering, Central South University, Changsha, Hunan 410083, China

[*] Corresponding authors. Phone: 86-731-8887 9616.
E-mail addresses: dengliu@csu.edu.cn (L. Deng); liuhuixia401973@csu.edu.cn; (H. Liu); liuyounian@csu.edu.cn (Y.-N. Liu)

## Materials and Methods

### *Materials*

Tetraethyl orthosilicate (TEOS), resorcinol, ethanol, aluminum nitrate nonahydrate [$Al(NO_3)_3 \cdot 9H_2O$], streptozotocin (STZ), indocyanine green (ICG), (3-aminopropyl) triethoxysilane (APTES) and 1,3-diphenylisobenzofuran (DPBF) were purchased from Aladdin (Shanghai, China). Lysostaphin S10039 (Ly) was obtained from Yuanye Biotechnology (Shanghai, China). (3-Aminopropyl) triethoxysilane was obtained from Sigma-Aldrich (Shanghai, China). Live/Dead Bac Light bacterial viability kit was obtained from Invitrogen (USA). Staphylococcus aureus UA300 and Escherichia coli (*E. coli*) CMCC(B) 44102 were acquired from the Microbiology Conservation Centre (Shanghai, China). The tryptic soybean peptone liquid medium (TSB), and tryptic soybean peptone agar medium (TSA) were procured from Zhongshan Biowei (Guangzhou, China). Human umbilical vein endothelial cells (HUVEC) and mouse embryo fibroblast cells (NIH 3T3) were provided by Xiangya Hospital of Central South University (Changsha, China).

### *Characterization of nanoparticles*

The surface morphology of CSIL nanomotor was observed by scanning electron microscopy (SEM, JSM-7610F Plus, JEOL, Japan). The intrinsic morphology and size were displayed by transmission electron microscopy (TEM, Helios 5 CX, Thermo Fisher, USA). The size distribution and zeta potential were determined by Malvern Zetasizer (Nano ZS, Malvern, UK).

### *Measurement of the generated $^1O_2$*

DPBF solution (1mg $mL^{-1}$, 50 μL) was added into ethanol (50%, 2.9 mL). Then, CSI and CSIL (12 mg $mL^{-1}$, 50 μL) were added to the above solution. Under the NIR irradiation at 808 nm, the absorbance of the mixed solution at 410 nm was recorded every minute.

### *Evaluation of photothermal effects*

Photothermal performance of the CSILs were detected. At first, CS, CSI, CSIL (200 μg $mL^{-1}$, 1 mL) were placed in 2 mL bottles. Under the NIR irradiation with varied power densities (0.3, 0.6, 0.9 and 1.2 W $cm^{-2}$), the temperature variation was recorded at intervals of 30 s by the infrared thermography (FLIR C2, USA) until the temperature levels off. Secondly, the photothermal properties of CS, CSI, CSIL with various concentration gradients (50, 150 and 200 μg $mL^{-1}$) was evaluated. Thirdly, the

photothermal stability was investigated by irradiation of CS, CSI, CSIL (200 μg $mL^{-1}$) for 10 min at 1.2 W $cm^{-2}$. Then switched off the NIR laser, repeated the five cycles of "On-Off" process.

***Evaluation of targeting properties of CSIL nanomotor toward MRSA***

To investigate the target properties of CSIL to MRSA, MRSA and *E*. coli were stained by STOY9 (green fluorescence) for 0.5 h. Then, the bacteria suspension was washed to remove the unstained bacteria. Next, rhodamine B (red fluorescence) labeled CSIL was co-cultured with MRSA and *E. coli* for 1 h, respectively. The above suspensions were centrifuged at 600 rpm to remove the uncombined CSIL. Finally, the contact ratios of red and green fluorescence were measured using confocal laser microscopy.

The Y-shaped channel consists of a main channel measuring 1.0 cm in length and 0.4 cm in width, and branch channels measuring 0.8 cm in length and 0.3 cm in width. First, hydrogels were implanted in chambers (ii) and (iii) to prevent bacteria from retrograde beforehand and then MRSA and *E*. coli ($10^8$ CFU $mL^{-1}$) was cultivated in chambers (ii) and (iii), separately. After adding 50 μL of rhodamine B labeled CSIL solution dispersed in PBS to the reservoir (i), and exposed to the NIR light. The red fluorescence in the channel and the fluorescence intensity images in reservoirs (ii) and (iii) was observed using confocal fluorescence microscopy.

***Biocompatibility test***

Fresh blood of mouse was collected and centrifuged at 1000 rpm for 5 min, and then washed 5 times with PBS. The erythrocyte solution was blended with various concentrations of CSIL and incubated in a 37 °C water bath for 12 h, deeming deionized water and PBS as positive and negative controls. Subsequently, the supernatant (100 μL) was taken, and measured the absorbance at 559 nm by enzyme marker (Bio-Tek ELx800, USA). The hemolysis percentage was then calculated.

To clarify the impact of CSIL to normal cells, HUVEC and NIH 3T3 were cultured in DMEM culture medium, with a seeding density of $10^4$ cells per well in a 96-well plate. After incubation for 24 h, the same concentration gradient of CSIL applied in hemolysis test was introduced. Further 24 h of co-incubation processed, CCK-8 reagent was administrated into all wells, and the absorbance of the supernatant at 450 nm was measured by the microplate reader after 0.5 h incubation.

***Crystalline violet staining of MRSA biofilms***

The biofilm with different treatments were adhibited to staining with a 0.1% solution of crystalline violet for 15 min, followed by three gentle times rinse using PBS. In the next, we dissolved biofilm samples with 30% acetic acid and assessed the absorbance at a wavelength of 559 nm.

**Morphology of MRSA observed by SEM**

MRSA and *E. coil* were planted on silicon wafer, and the administration procedures were the same as antibacterial *in vitro*. The immobilization of bacteria on the wafer was achieved by overnight treatment with 4% glutaraldehyde, followed by dehydration using varied concentrations of ethanol for 20 min. Ultimately, the morphology of MRSA was observed by SEM.

***Observation of MRSA biofilm morphology under CLSM***

Live/Dead BacLight Bacterial Survival kit was used to dye the bacterial. Similarly, 10 min of NIR light exposure after drug administration. Image with CLSM, the green fluorescent and red fluorescent was classified as live and dead bacterial, separately.

***Statistical analysis***

In our research, all data were expressed as mean ± standard deviation. Student's t-test and one-way ANOVA test were applied for two groups and multiple group comparisons respectively. Significant differences were presented as $*p < 0.05$, $**p < 0.01$, and $***p < 0.001$, and ns denotes no statistically significant.

## Supplemental Figures and Tables

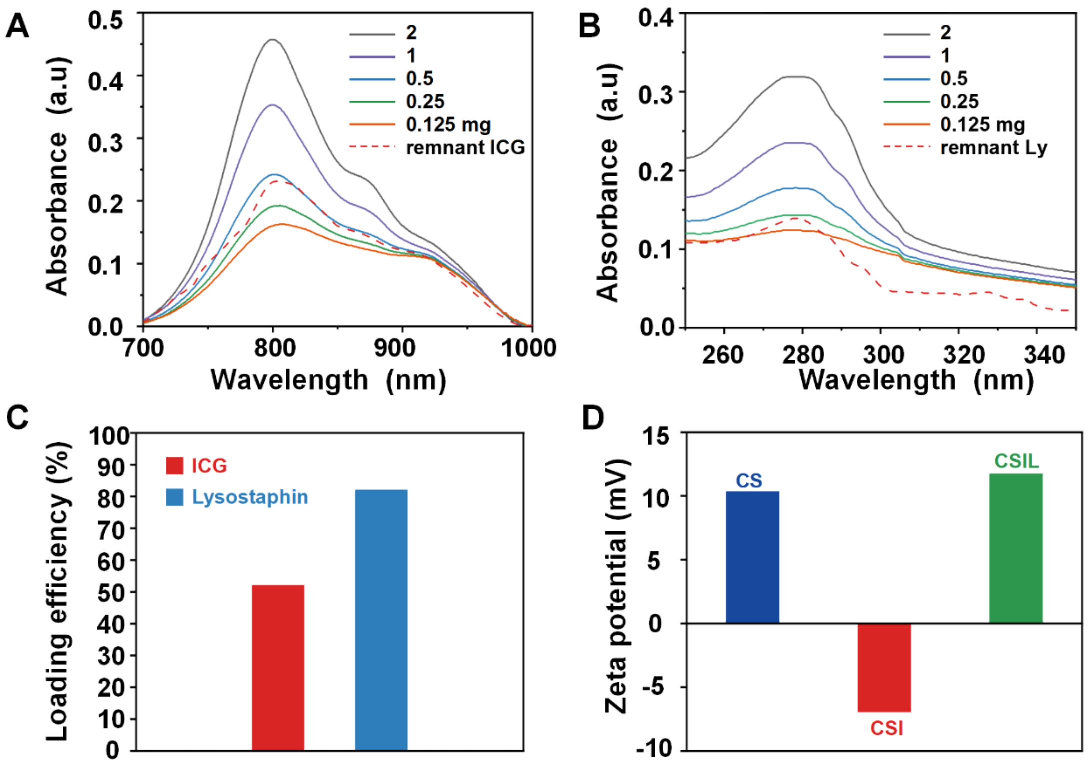


**Figure S1**. Loading properties of ICG and lysostaphin on nanomotors. (A) UV-vis spectra of the different concentrations ICG and the remnant ICG in the solution with CSIL nanomotor. (B) UV-vis spectra of the different concentrations lysostaphin and the remnant lysostaphin of CSIL in the solution with CSIL nanomotor. (C) Loading efficiency of ICG and lysostaphin. (D) Zeta potentials of the nanoparticles.

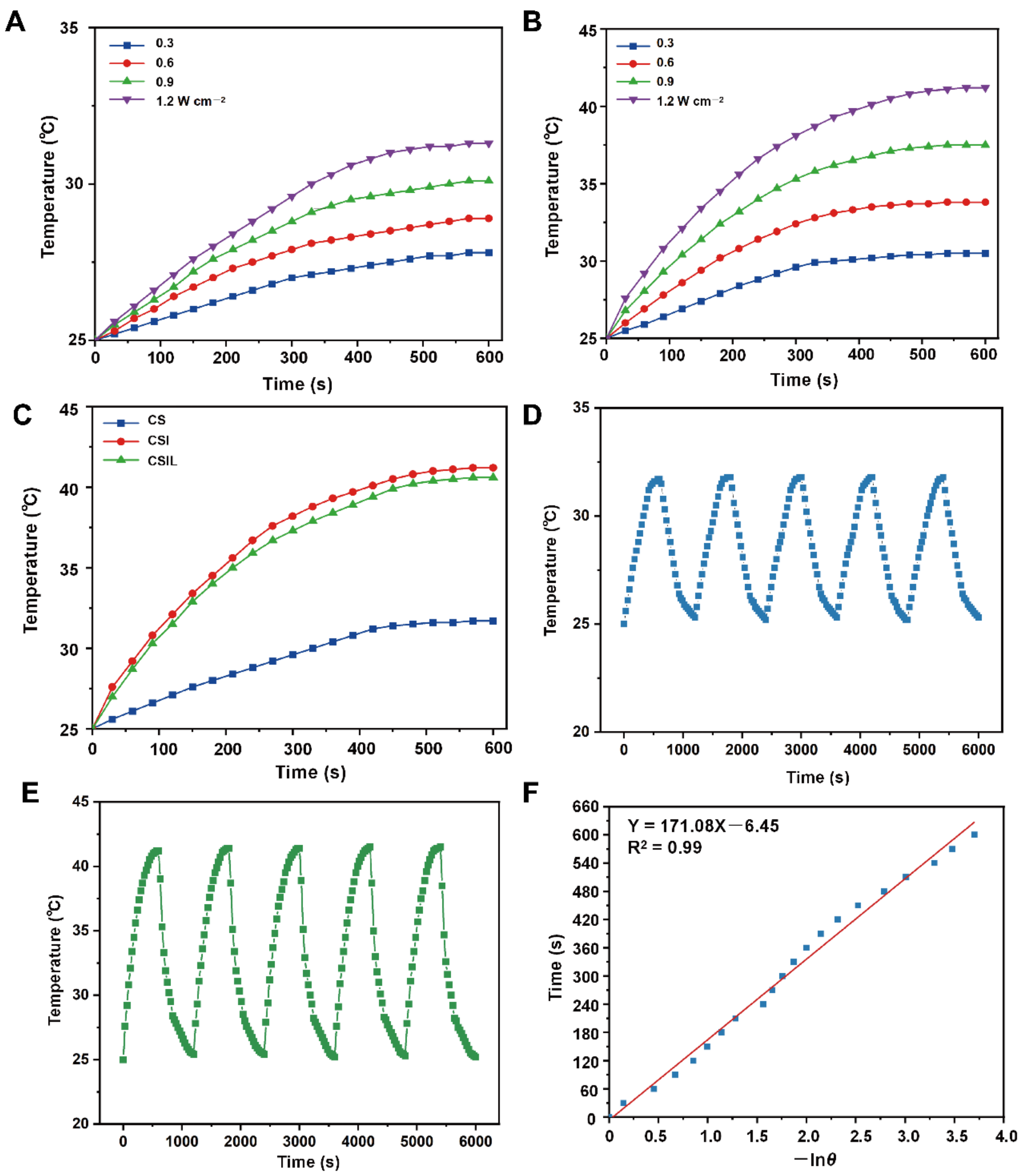


**Figure S2**. (A) Changes in temperature rise over time on CS (200 μg mL$^{-1}$) under different NIR power density. (B) Changes in temperature rise over time on CSI (200 μg mL$^{-1}$) under different NIR power density. (C) Changes in temperature rise over time on CS, CSI and CSIL (200 μg mL$^{-1}$) under NIR irradiation (1.2 W cm$^{-2}$). (D) Recycling heating fluctuation of CS (200 μg mL$^{-1}$) under NIR irradiation (1.2 W cm$^{-2}$) for five on/off cycles. (E) Recycling heating fluctuation of CSI (200 μg mL$^{-1}$) under NIR irradiation (1.2 W cm$^{-2}$) for five on/off cycles. (f) The relation between irradiation time and $-\ln\theta$.

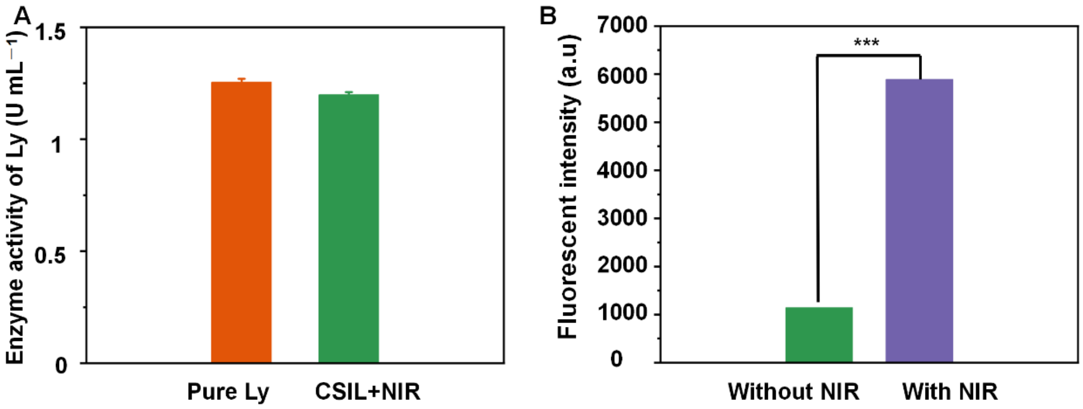


**Figure S3.** (A) Comparision of the enzyme activity between Ly alone and Ly in CSIL+NIR group. (B) Fluorescence intensity of Rhodamine B-dyed CSIL in the low chamber with or without NIR irradiation, ***p < 0.001.

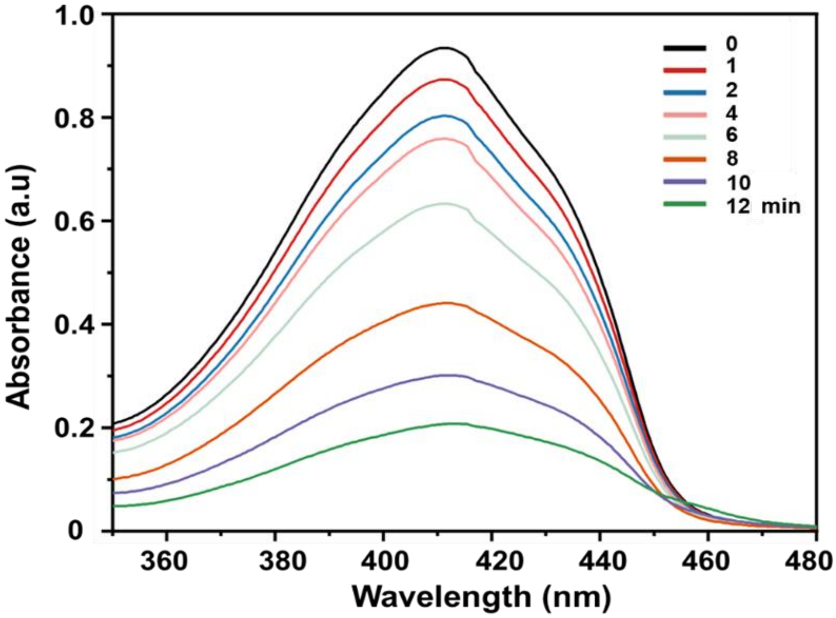


**Figure S4**. Absorbance of DPBF in group CSI+NIR at varied time.

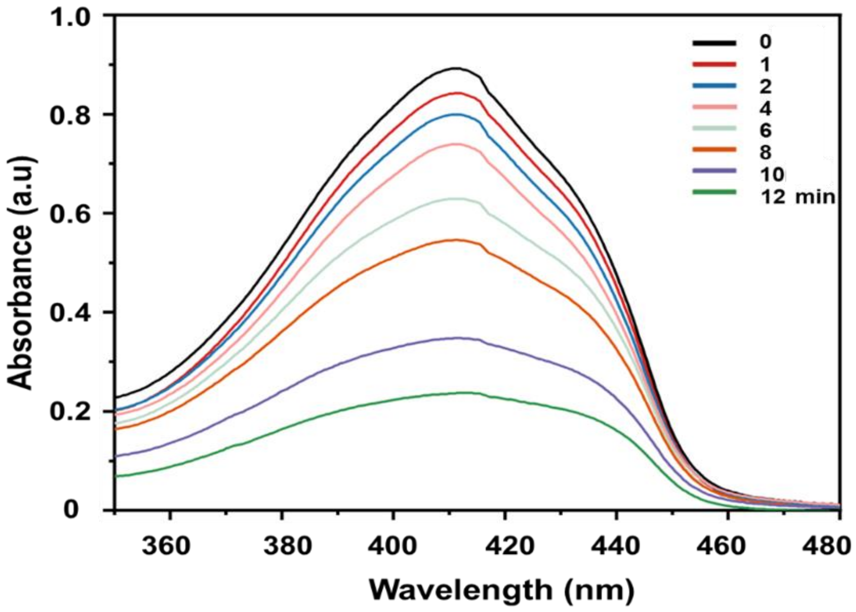


**Figure S5**. Absorbance of DPBF in group CSIL+NIR at different time.

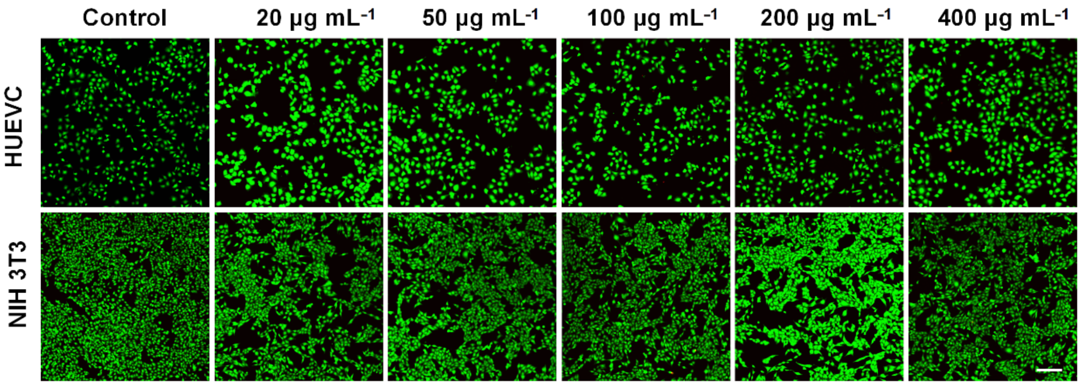


**Figure S6**. Fluorescence photographs of live/dead staining of HUVEC and NIH 3T3 under different concentrations of CSIL. Scale bars: 200 μm.

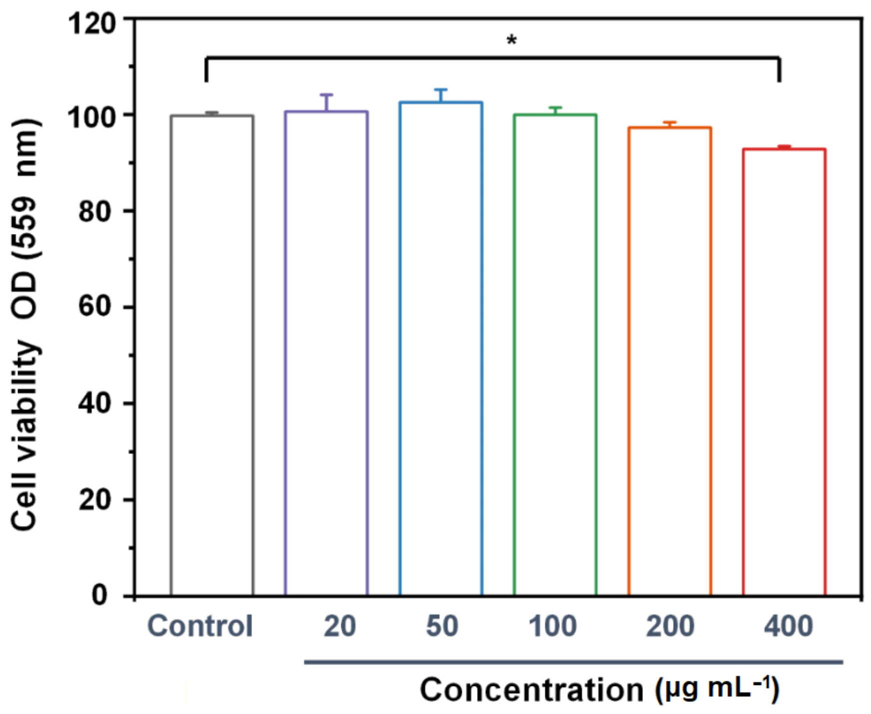


**Figure S7**. Cytotoxicity of different concentrations of CSIL for HUVEC, *p < 0.001.

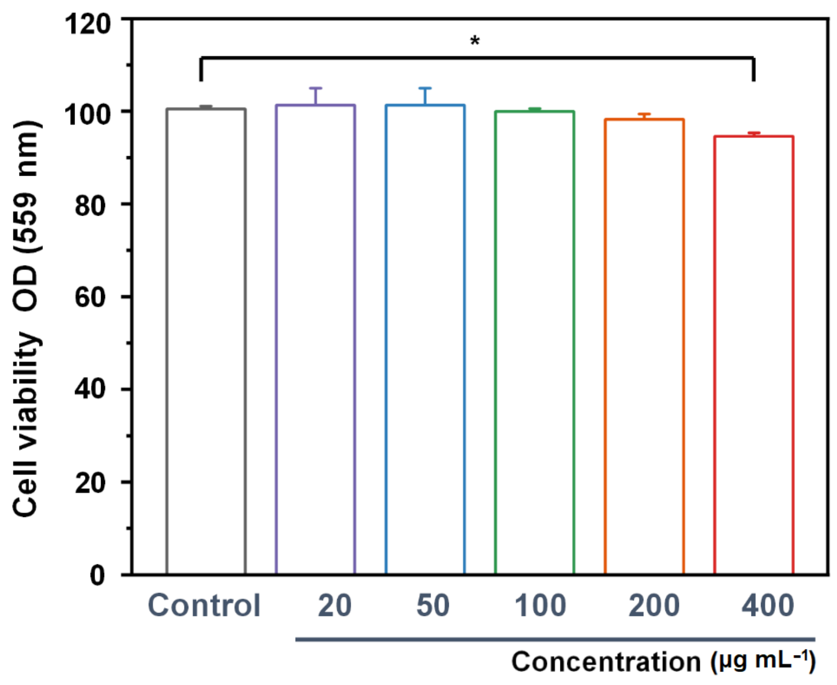


**Figure S8**. Cytotoxicity of different concentrations of CSIL for NIH 3T3, *p < 0.001.

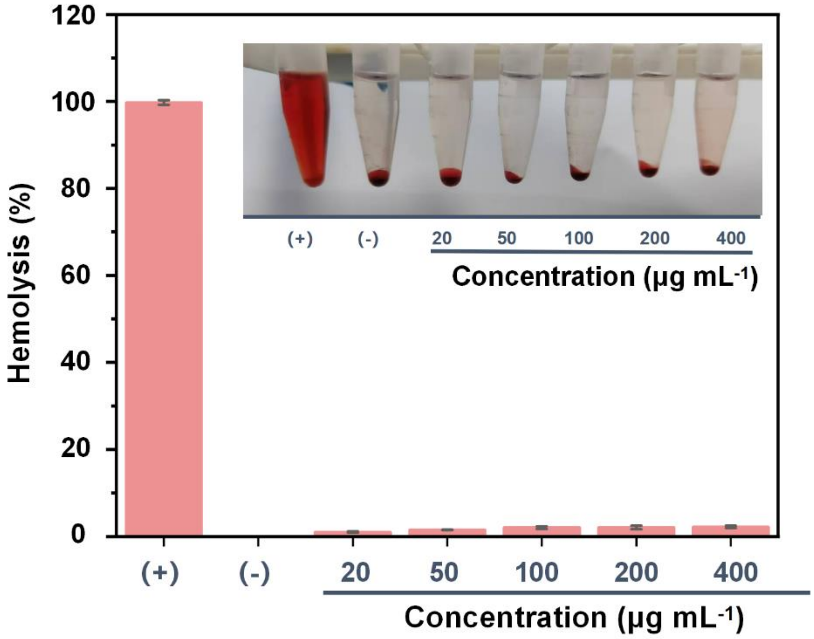


**Figure S9**. Hemolysis rate of CSIL at different concentrations.

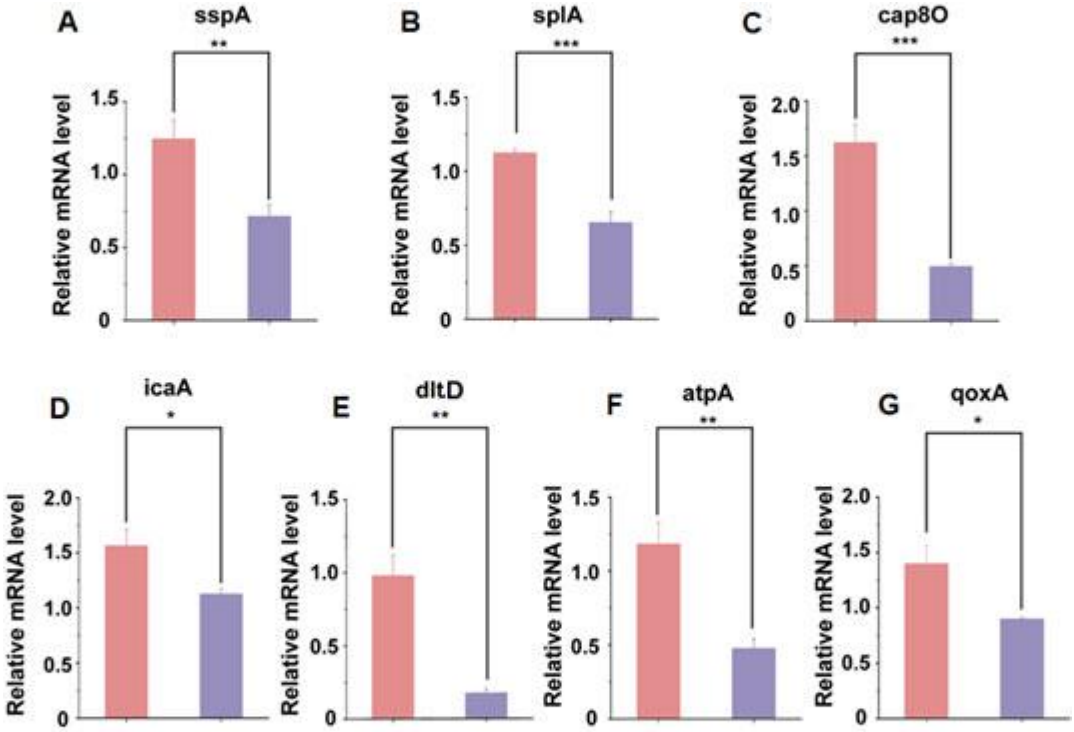


**Figure S10**. qPCR changes of MRSA after CSIL motor intervention. (A) The sspA gene; (B) splA gene; (C) The cap8O gene; (D) icaA gene; (E) dltA gene; (F) atpA gene; (G) qoxA gene.Pink:control group; Purple : CSIL+NIR group.

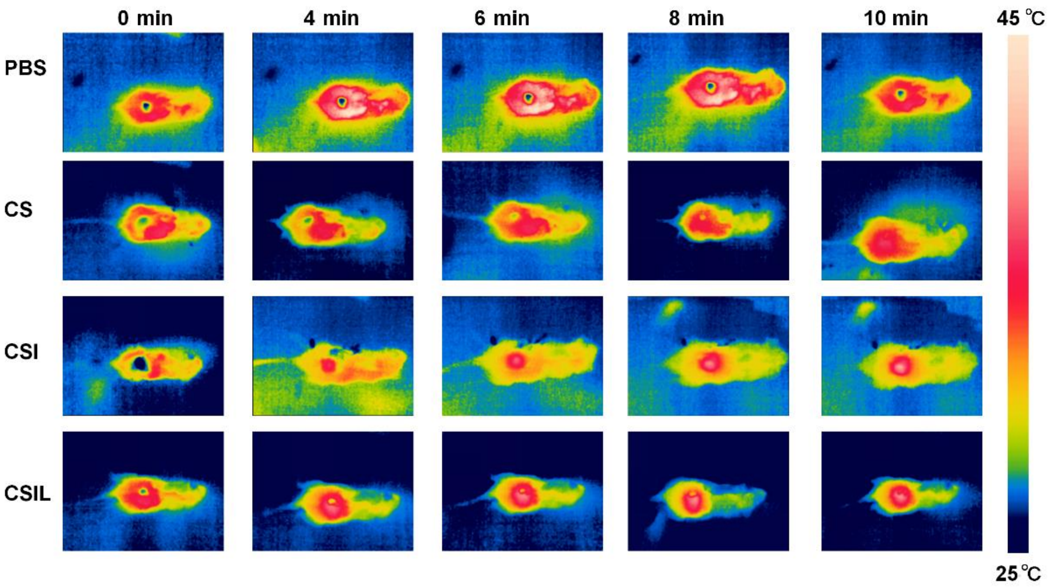


**Figure S11**. Infrared thermal images of mice after different treatments.

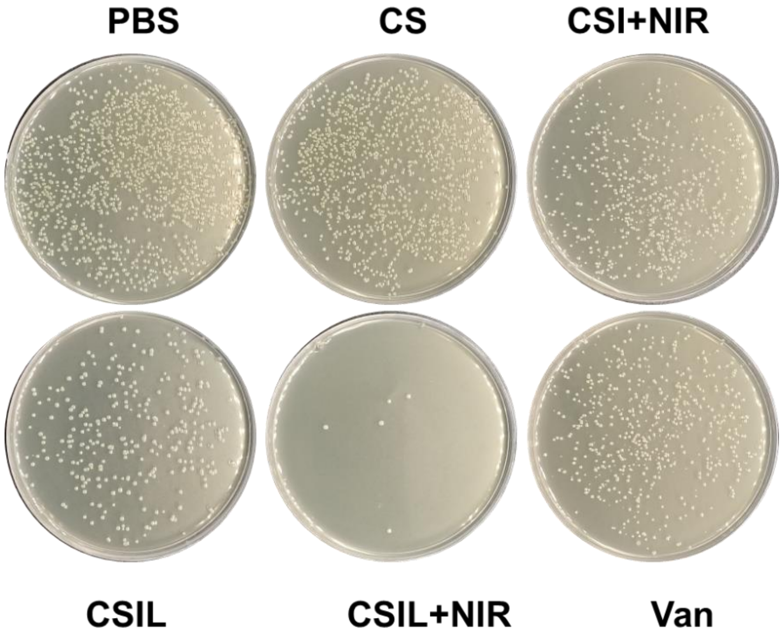


**Figure S12**. Representative plates of bacterial colonies isolated from the wounds after 16 days treatment *in vivo*.

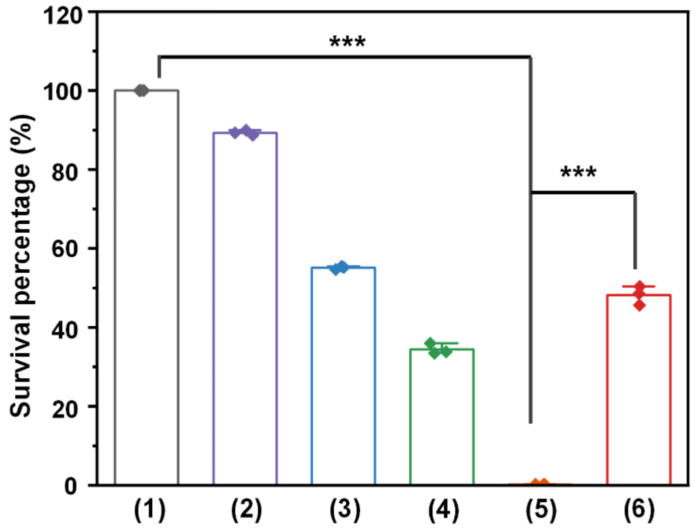


**Figure S13**. Survival percentage of MRSA in the wounds of (1) PBS, (2) CS, (3) CS+NIR, (4) CSIL, (5) CSIL+NIR and (6) Van group at day 16.

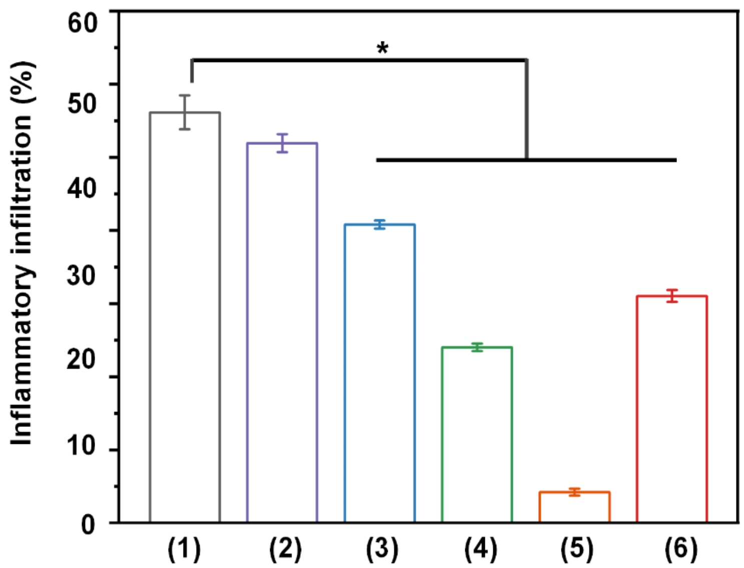


**Figure S14**. Inflammatory infiltration percentage in the wounds of (1) PBS, (2) CS, (3) CS+NIR, (4) CSIL, (5) CSIL+NIR and (6) Van group at day 16.

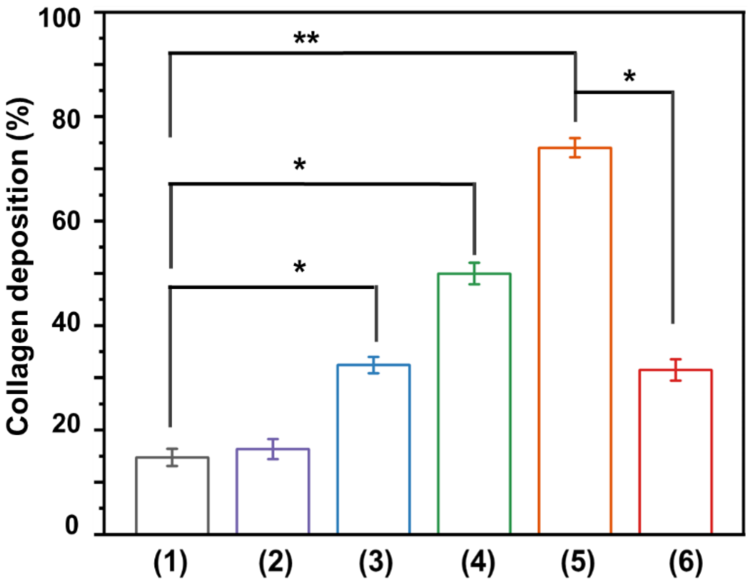


**Figure S15**. Collagen deposition percentage in the wounds of (1) PBS, (2) CS, (3) CS+NIR, (4) CSIL, (5) CSIL+NIR and (6) Van group at day 16.

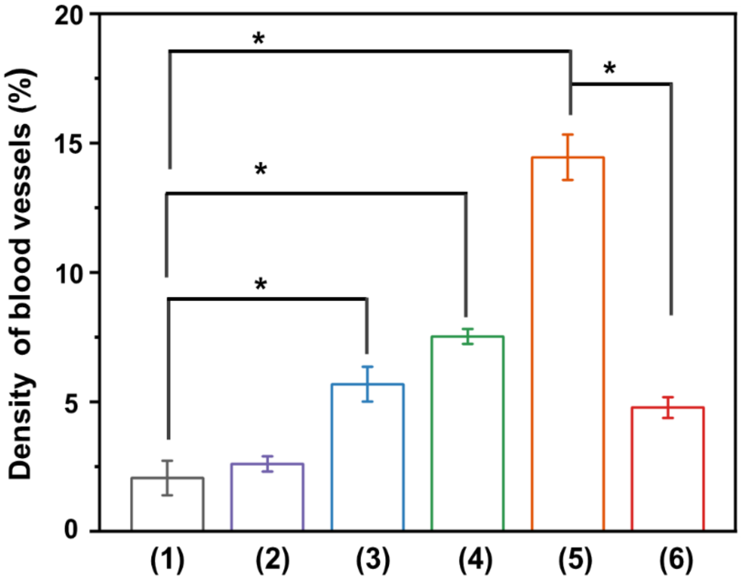


**Figure S16**. Blood vessels density in the wounds of (1) PBS, (2) CS, (3) CS+NIR, (4) CSIL, (5) CSIL+NIR and (6) Van group at day 16.

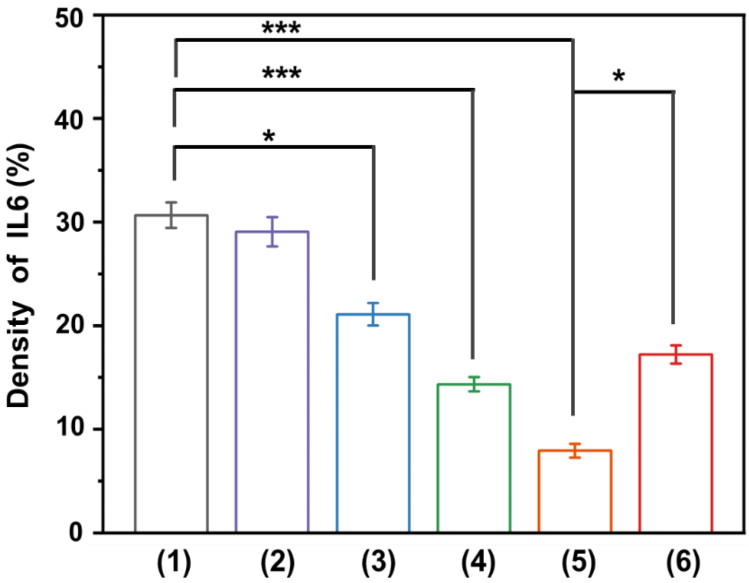


**Figure S17**. Area of IL-6 positive area of wounds in (1) PBS, (2) CS, (3) CS+NIR, (4) CSIL, (5) CSIL+NIR and (6) Van group at day 16.

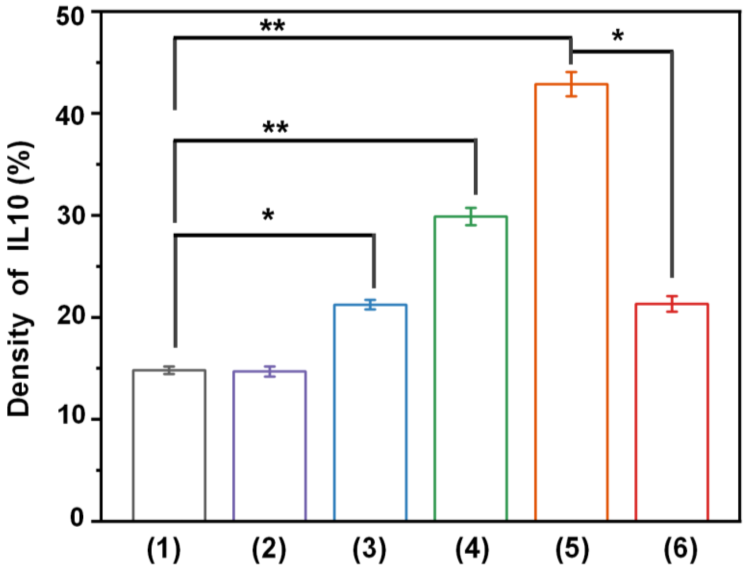


**Figure S18**. Area of IL-10 positive area of wounds in (1) PBS, (2) CS, (3) CS+NIR, (4) CSIL, (5) CSIL+NIR and (6) Van group at day 16.

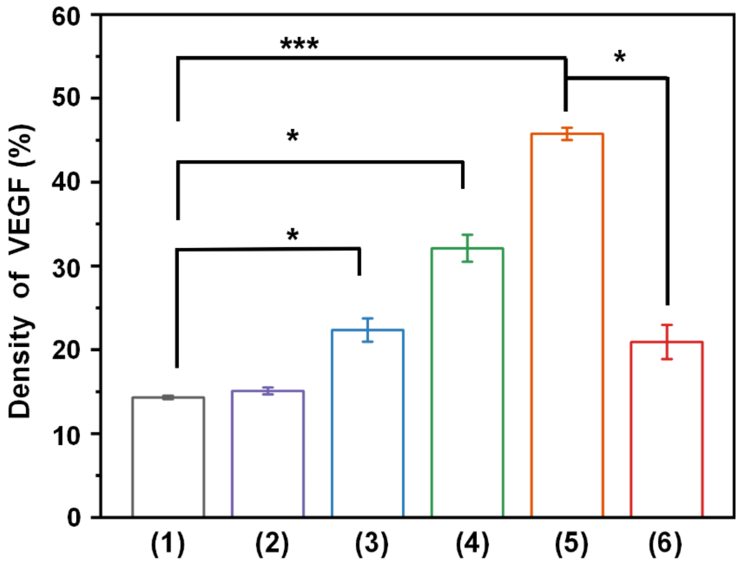


**Figure S19**. Area of VEGF positive area of wounds in (1) PBS, (2) CS, (3) CS+NIR, (4) CSIL, (5) CSIL+NIR and (6) Van group at day16.

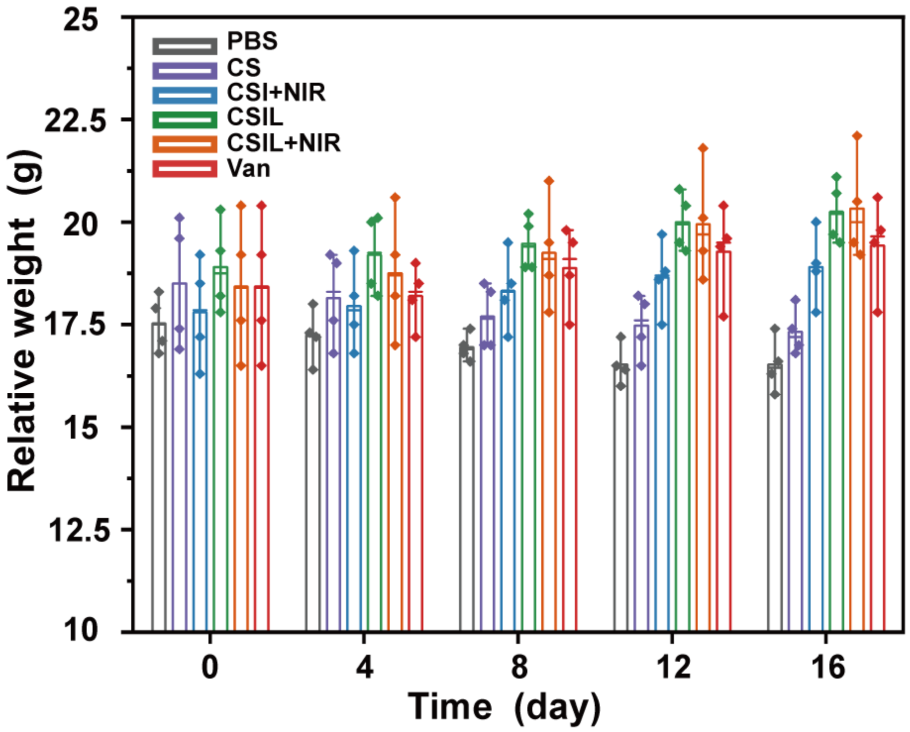


**Figure S20**. Change in body weight for each group (PBS, CS, CSI+NIR, CSIL, CSIL+NIR and Van) of mice.

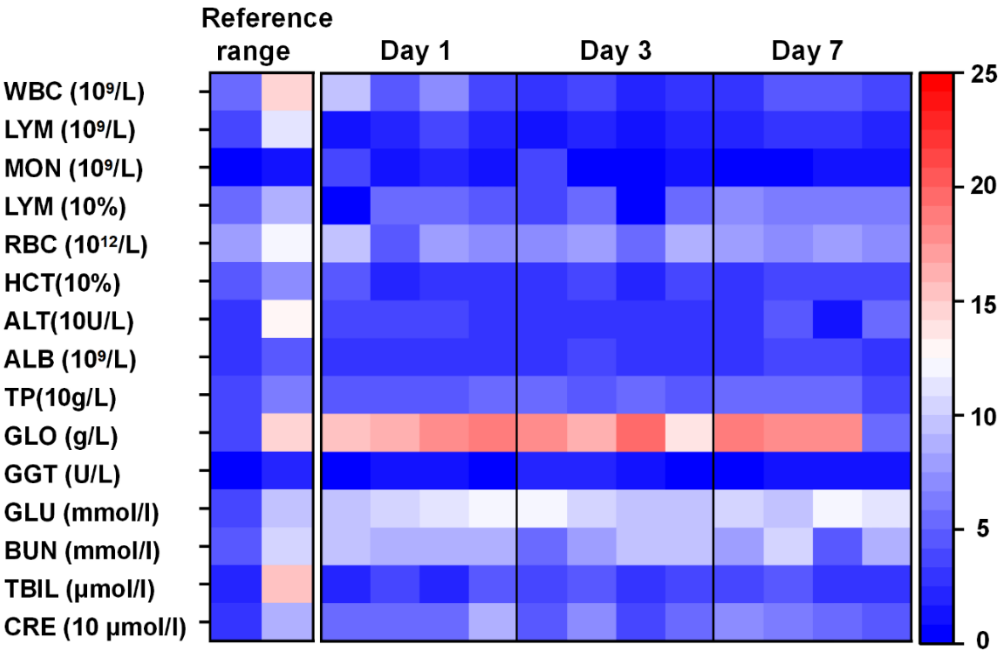


**Figure S21**. Blood cell numbers and liver & kidney function indicator in mice and after treatment in day 1, day 3 and day 7, respectively.

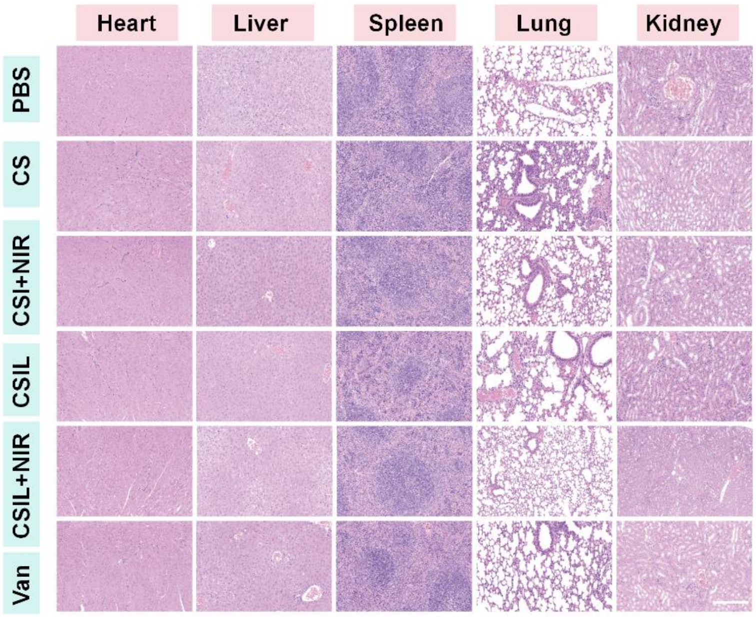


**Figure S22**. H&E staining of major organs collected from mice at 16 days post-treatment. Scale bars: 500 μm.

Table S1. Genetic primer information

| Gene | Genebank No | Primer (5’-3’) | Length |
|---|---|---|---|
| atpA | SAUSA300_RS11345 | F:AGGTATTACTGAAGGTGACGAAGTT<br>R: TAGCTTTTTTCTCTACTGGACGTGT | 161 |
| cap8O | SAUSA300_RS00875 | F: CGACAATTGCGCCTAAAACG<br>R: TCCCTGAACGAATGTGCGATAG | 218 |
| dltA | SAUSA300_RS04515 | F:AATCCACAAAGCATTGCTGTTAGAC<br>R: GGCACCAATCATCCCAACAA | 162 |
| icaA | SAUSA300_RS14455 | F:GATACGTTGTCTAATGTTCTTGCAC<br>R: TGAGTGCGTTGGCTTTACCT | 166 |
| qoxA | SAUSA300_RS05175 | F: GTTGCTGCTTTAGCTATTCCTACAG<br>R: GTTGCTGCTTTAGCTATTCCTACAG | 186 |
| sspA | SAUSA300_RS05110 | F：GCAACTTTGACAACAGCGACAC<br>R：TTGCTTGACTGCGTTTGTTG | 98 |
| splA | SAUSA300_RS09620 | F：TTCAGTGGTAGCATTTGTGGGTG<br>R:TGAACATGAACTATCGCAAGGTCTT | 107 |
